# Piezo1 as a mechanical checkpoint in T cell immunotherapy for solid tumors

**DOI:** 10.3389/fphar.2026.1722027

**Published:** 2026-02-09

**Authors:** Xinmu Cui, Jianan Zhao, Huajie Tian

**Affiliations:** 1 Department of Medical Technology, Changchun Medical College, Changchun, China; 2 Department of Cardiovascular Sciences, Temple University, Philadelphia, PA, United States; 3 Department of Hematology, Shuguang HospitaI Affiliated to Shanghai University of Traditional Chinese Medicine, Shanghai, China

**Keywords:** chimeric antigen receptor (CAR)-T cells, mechanoimmunology, Piezo1, solid tumors, T cell exhaustion, T cells, tumor microenvironment (TME)

## Abstract

T cell-based immunotherapies exhibit limited efficacy against solid tumors, a challenge primarily attributed to the immunosuppressive and mechanically hostile tumor microenvironment (TME). Within this context, the mechanosensitive ion channel Piezo1 has emerged as a key TME mechanosensor, yet its role in modulating T cell-mediated anti-tumor immunity remains to be fully elucidated. This review aims to synthesize existing evidence on Piezo1’s regulation of T cell functions, including activation, proliferation, and infiltration, and its broader impact on immunotherapy for solid tumors. We highlight Piezo1’s dual regulatory function in the immune landscape: acute activation robustly enhances T cell effector functions and cytotoxicity, whereas chronic stimulation within the stiff TME paradoxically promotes T cell exhaustion. Importantly, preclinical studies demonstrate that modulating Piezo1 signaling, particularly in combination with matrix normalization synergistically enhances the infiltration, persistence, and overall antitumor efficacy of adoptive T cells and endogenous immune responses. These findings position Piezo1 as a promising mechanical checkpoint for improving T cell therapies. Nevertheless, significant challenges persist for clinical implementation, including the heterogeneity of mechanical signals and the pleiotropic nature of Piezo1 across different cell types. Future research should therefore focus on developing T cell-specific mechanotherapies, identifying novel targets, and validating mechanical biomarkers to guide patient stratification, thereby accelerating the clinical translation of “mechanoimmunology”.

## 1 Introduction

Cancer has emerged as a significant global health burden. The tumor microenvironment (TME) profoundly regulates the initiation, progression, and metastasis of malignant tumors (Siegel et al., 2023; de Visser and Joyce, 2023). Far from being solely composed of tumor cells, the TME is a highly complex and dynamic ecosystem comprising diverse elements, including stromal cells, signaling molecules, and non-cellular components. In solid tumors, aberrant mechanical properties constitute a prominent feature of the TME, characterized by excessive extracellular matrix (ECM) deposition and stiffening, elevated interstitial fluid pressure (IFP), and the accumulation of solid stress (Deng et al., 2022). These abnormal mechanical signals are not only passive physical barriers, but actively drive tumorigenesis, disease progression, and resistance to therapy by regulating key cellular behaviors such as proliferation, invasion, and metastasis, and modulating key signaling pathways such as integrin and Yes-associated protein/Transcriptional co-activator with PDZ-binding motif (YAP/TAZ) (Pavel et al., 2018; Zanconato et al., 2016).

Although T cell-based immunotherapies, including Chimeric Antigen Receptor (CAR)-T cells and T cell receptor (TCR)-engineered T cells, have demonstrated groundbreaking efficacy in hematologic malignancies and are hailed as a milestone in immunotherapy, their clinical benefits in solid tumors remain far below expectations (Albelda, 2024; Qi et al., 2025). The core reason for this significant gap lies in the complex and formidable multiple barriers constructed by the tumor microenvironment (TME) in solid tumors. Beyond established factors such as immunosuppressive cytokine networks, extensive infiltration of immunosuppressive cells, and high tumor antigen heterogeneity (Nia et al., 2020; Chen et al., 2023), the inherent abnormal biomechanical properties of the TME are increasingly recognized as severely impeding T cell infiltration, persistence, and ultimate cytotoxic function. ECM stiffness, elevated IFP, and solid stress have become critical physical and functional barriers limiting the efficacy of adoptive T cell therapies (ACT) and endogenous anti-tumor immunity (Davenport et al., 2018; Greiner et al., 2023). These mechanical cues drive tumor cell proliferation, invasion, and metastasis while directly impairing the normal function of immune cells. Moreover, the rigid physical environment can indirectly compromise the cytotoxic activity of immune cells by modulating their signal transduction and functional states (Shi et al., 2022; Liu et al., 2025). This mechanical resistance is particularly pronounced within highly desmoplastic and fibrotic solid tumors, notably pancreatic ductal adenocarcinoma (PDAC) and triple-negative breast cancer (TNBC). It is closely associated with poor prognosis and treatment resistance (Barbazan et al., 2023; Angeli et al., 2025).

Within the tumor microenvironment (TME), the intricate network through which tumor-infiltrating immune cells perceive and respond to mechanical forces involves multiple mechanisms, with the mechanosensitive ion channel Piezo1 playing a central mediating role (Nikoo et al., 2024; Jung et al., 2023). Piezo1, a widely distributed key mechanosensor, efficiently and precisely converts external physical stimuli, such as matrix stiffness and pressure, into intracellular ion flux and a series of biochemical signals (Lai et al., 2022). Recent research confirms that Piezo1 activation by mechanical stress in the TME is a critical step in regulating T cells biological behavior, significantly impacting T cells’ tumor migratory capacity, activation status, and the formation and stability of immunological synapses—an essential structural basis for effective target cell recognition and killing of target cells (Schurich et al., 2019; Qu and Zhang, 2025; Greiner et al., 2023). However, while numerous reviews discuss the failure of immunotherapies in solid tumors, they predominantly focus on biochemical barriers, such as chemical signals and immunosuppressive cells, often overlooking the critical dimension of the tumor microenvironment’s (TME) physical biomechanics (Smith and Kang, 2013; Jeffreys et al., 2024). Similarly, despite the burgeoning research on Piezo1, a comprehensive review that systematically links Piezo1-mediated mechanotransduction to T cell dysfunction, acting as a “mechanical checkpoint,” is currently lacking (Hope et al., 2022). Therefore, dissecting the role of Piezo1-mediated mechanotransduction in regulating the function of T cells is a crucial prerequisite for overcoming the therapeutic bottleneck in solid tumors (Zhuang et al., 2023; Pang et al., 2024). This review aims to elucidate three key aspects: first, to systematically clarify the core role of aberrant biomechanical properties within the TME in limiting the efficacy of T cell immunotherapy for solid tumors (Anonymous, 2024); second, to deeply analyze the complex role of Piezo1 mechanosignaling in regulating T cell function and its potential dual effects on immune fitness (Bonner et al., 2025). Moreover, third, to explore the translational prospects and future research directions for targeting such mechanosensing and transduction pathways, with Piezo1 as a primary representative, to overcome immunosuppression in solid tumors, thereby offering potential breakthroughs for the emerging field of mechano-immunology (Abiff et al., 2023).

## 2 Mechanical properties of the TME and their dual regulation of T cells

### 2.1 Tumor microenvironment: a mechanically remodeled space

**FIGURE 1:**
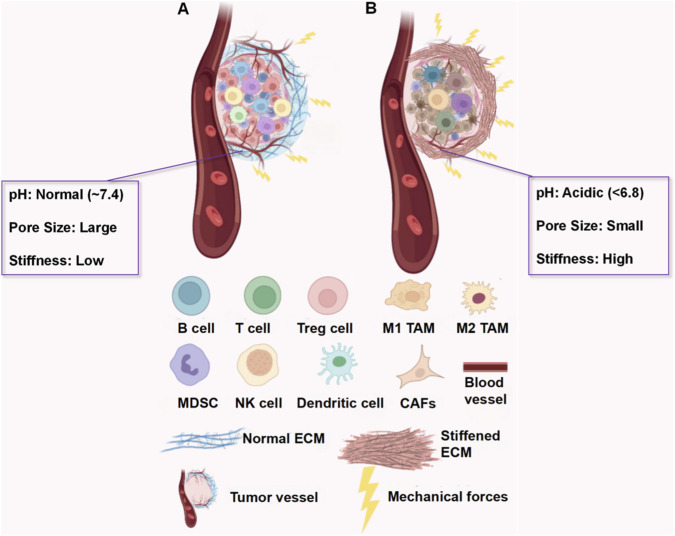
The comparative effects of normal versus stiffened extracellular matrix (ECM) on tumor immune infiltration in the tumor microenvironment (TME) and the associated mechanical forces. ECM density is distinguished by fiber quantity (sparse in normal ECM vs. dense in stiffened ECM). **(A)** Normal ECM: It is characterized by high flexibility and low mechanical stiffness and forces, a physiological pH (∼7.4), and large pore size, enabling unimpeded infiltration of immune cells into the tumor core. **(B)** Stiffened ECM: It is featured by rigidity, pro-fibrotic properties, and a marked increase in mechanical stiffness and forces, an acidic pH (<6.8), and small pore size, forming a robust physical barrier that restricts immune cell infiltration.

The ECM serves as a key structural component and the primary driver of mechanical remodeling within the TME (Mai et al., 2024). Its physical properties, such as stiffness and crosslinking density, not only directly affect the tumor’s mechanical microenvironment and the transduction of mechanical forces within it, but also regulate the tumor’s immune response (Yang et al., 2024). Specifically, the properties of the ECM exert diametrically opposite effects on the infiltration of tumor immune cells. On one hand, the loose and structurally normal ECM (characterized by low cross-linking, loose architecture and a physiological stiffness of <1 kPa in human normal soft interstitial tissues) facilitates immune cell infiltration and eliminates physical barriers (Kuczek et al., 2019; Discher et al., 2005). On the other hand, the stiffened and highly crosslinked extracellular matrix (ECM) in human malignant solid tumors forms a dense fibrous physical barrier that not only restricts immune cell migration via the physical property of reduced pore size, but also displays significantly elevated mechanical stiffness, which enhances mechanical resistance to migration and thereby synergistically suppresses the infiltration of immune cells including T cells and natural killer (NK) cells (Zhao et al., 2021; Tabdanov et al., 2021; Ai et al., 2026; Wolf et al., 2013). Furthermore, the mechanical forces transmitted by the ECM can also regulate the function of tumor-resident cells; for instance, it can induce tumor-associated macrophages (TAMs) to secrete a variety of immunosuppressive cytokines, thereby establishing an immunosuppressive microenvironment and suppressing the antitumor activity of immune cells (Biray Avci et al., 2024). Taken together, existing studies have confirmed that stiffened ECM not only physically restricts the infiltration of T cells and NK cells by exerting strong mechanical resistance, but also impairs their cytotoxic function. Multiple mechanisms act together, resulting in a significant impairment of the antitumor immune response (Figure 1).

### 2.2 Advances in mechanics of T cell therapy targeting the TME

CAR-T cell therapy has demonstrated remarkable efficacy in the treatment of hematologic malignancies, achieving favorable complete response rates in acute lymphoblastic leukemia and diverse lymphoma subtypes (Eyquem et al., 2017). CAR-T cells represent a pivotal prototype of T cell-based immunotherapies, and their limited efficacy in solid tumors reflects a broader challenge faced by most therapeutic T cell subsets. Despite these promising outcomes, their effectiveness against solid tumors remains limited, with clinical response rates typically below 30% (Hou et al., 2019). This discrepancy is primarily attributed to the immunosuppressive and biomechanically hostile TME, which poses significant obstacles to therapeutic T cells’ infiltration and function. The mechanical properties of the TME in solid tumors are complex and variable, posing multiple challenges to the function of T cells. The specific impacts and their associations with the potential regulation of Piezo1 are shown in Table 1. A key feature of the solid TME is its altered mechanical properties, including ECM stiffening, aberrant fluid dynamics, and elevated solid stress (Liu et al., 2022). These elements collectively establish a physical barrier that severely restricts T cells’ mobility and antitumor activity (Albelda, 2024; De Vecchis et al., 2021). In highly stromal-like tumors such as pancreatic and breast cancers, cancer-associated fibroblasts (CAFs) drive excessive deposition and crosslinking of extracellular matrix components, particularly type I collagen. This results in tissue stiffness with shear moduli an order of magnitude higher than those in normal tissues (Nia et al., 2016; Masuda, 2025). The consequent reduction in interstitial pore sizes to less than 5 μm physically impedes the migration of CAR-T cells, which typically measure 7–10 μm in diameter. As a result, CAR-T cells are often trapped in the peripheral stromal regions, leading to minimal penetration into the tumor core. Beyond structural constraints, the TME also exerts potent immunosuppressive biochemical pressures. Specifically, elevated levels of immunosuppressive cytokines, such as transforming growth factor-β (TGF-β) and interleukin-10 (IL-10), can promote the upregulation of co-inhibitory receptors on T cell surfaces, including programmed death-1 (PD-1) and lymphocyte activation gene 3 (LAG-3) (Wu B. et al., 2023). This, in turn, contributes to T cell exhaustion and subsequent functional impairment (Wherry and Kurachi, 2015; Chow et al., 2022). Additionally, extracellular acidification, with pH levels often dropping below 6.8, significantly impairs the metabolic activity and cytotoxicity of T cells (Corbet and Feron, 2017). Meanwhile, as detailed in Table 1, these mechanical stresses also disrupt vascular and lymphatic function (Wang et al., 2023), leading to high IFP, which further opposes T cell infiltration.

**TABLE 1:** Mechanical properties of the solid TME: Implications for T Cell dysfunction and Piezo1-Mediated regulation.

Mechanical property	Parameter (tumor vs. Healthy)	Impact on T cells	Piezo1-mediated mechanism	Clinical strategy	References
ECM Stiffness	Malignant tumors (e.g., breast cancer): several kPa; Normal soft tissues: <1 kPa	Infiltration↓ Cytotoxicity↓	Ca^2+^↑ → YAP/TAZ↑ → PD-1/LAG-3↑ IFN-γ/PRF1↓	Assess: MRE Intervene: Piezo1 antagonist + PD-1/LAG-3 inhibitor	(Acerbi et al., 2015; Plodinec et al., 2012; Levental et al., 2009; Maneshi et al., 2021)
IFP	10–40 mmHg vs. 0–2 mmHg	Migration↓ (physical barrier)	IFP↑→ECM densification → Prolonged Piezo1 activation	Assess: CEUS/CT Intervene: Vascular normalizers + Piezo1 modulators	(Heldin et al., 2004; Salavati et al., 2022)
Solid Stress	Exponential↑ vs. Low	Abnormal morphology; Migration↓; Unstable immune synapse	ERK-WASP↑ → Actin over-polymerization TGF-β↑ → YAP/TAZ↑ → Immunosuppression	Assess: Elastography Intervene: ERK/WASP inhibitor + Piezo1 modulators	Kumari et al. (2023)

CEUS, Contrast-Enhanced Ultrasound; CT, computed tomography; Ca^2+^, Calcium ion; ECM, extracellular matrix; ERK, Extracellular Signal-Regulated Kinase; IFN-γ, Interferon-gamma; IFP, interstitial fluid pressure; LAG-3, Lymphocyte-Activation Gene 3; MRE, magnetic resonance elastography; PD-1, Programmed Death-1; PRF1, Perforin-1; TAZ, Transcriptional Co-activator with PDZ-binding Motif; TGF-β, Transforming Growth Factor-beta; TME, tumor microenvironment; WASP, Wiskott-Aldrich Syndrome Protein; YAP, Yes-associated Protein.

Notably, mechanical and biochemical cues are interlinked through mechanotransduction pathways (Jung et al., 2025). Mechanosensitive ion channels, particularly Piezo1, play a crucial role in converting physical stimuli into biochemical responses within T cells. Acute activation of Piezo1 supports the formation of immunological synapses and enhances cytotoxicity, whereas chronic stimulation promotes T cell exhaustion (Pang et al., 2024; Liu et al., 2018). This dual role highlights the contextual influence of the TME on the function of CAR-T cells. Moreover, the differential activation of Piezo1 in hematological versus solid malignancies suggests distinct mechanistic adaptations across tissue types (Vasileva et al., 2021; Lebon et al., 2024; Yu and Liao, 2021). However, many mysteries remain in the research on the mechanism of interaction between Piezo1 and other mechanosensitive proteins. For example, the transient receptor potential vanilloid subtype 4 (TRPV4) and Piezo2, as well as on downstream effectors like YAP/TAZ—particularly regarding their tissue-specific mechanisms and pathological dynamic changes (Yang et al., 2022; Wang et al., 2019; Liang et al., 2022).

## 3 Piezo1-mediated mechanical signal transduction network: the molecular hub of T Cell function

**FIGURE 2:**
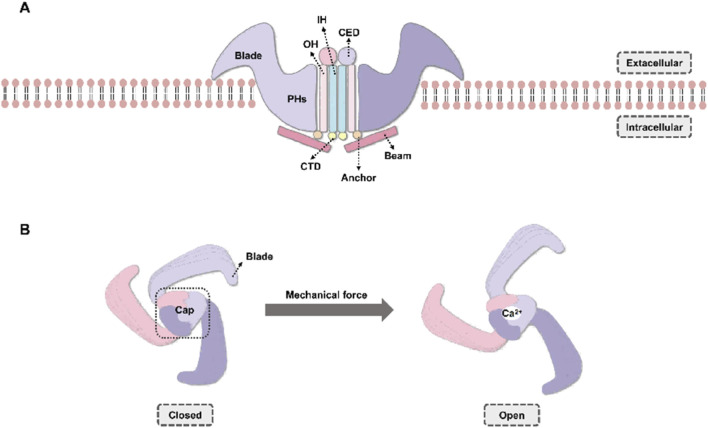
Structural Architecture and Mechanosensitive Gating of Piezo1. **(A)** Side view of the Piezo1 homotrimeric complex within the phospholipid bilayer. Key structural elements of the channel include: Blade domains, which are large, curved extracellular “propeller-like” structures primarily responsible for sensing and responding to membrane tension. Pore Helices (PHs): Helical segments that form the central ion-conducting pore and contribute to ion selectivity. Outer Helices (OHs): Transmembrane helices providing structural support and contributing to channel gating regulation. Inner Helices (IHs): Transmembrane helices situated on the inner side of the pore, constituting a critical component of the ion conduction pathway. They are sensitive to conformational changes induced by mechanical forces and are intrinsically involved in regulating channel gating. C-terminal domain (CTD): An intracellular domain that may link the channel to cytoskeletal anchoring sites. Beam/Anchor domain: An intracellular “lever-arm”-like structure that functions to transmit mechanical conformational changes to the pore region. **(B)** Top view illustrating Piezo1 mechanosensitive gating dynamics. Closed State (left panel): In the absence of mechanical stimulation, the Blade domains exhibit a highly curved conformation, which stabilizes the ion-conducting pore in a closed configuration (indicated by the dashed circle), thereby preventing ion permeation. Mechanical Activation (right panel): Upon the application of external mechanical forces (e.g., increased membrane tension or matrix stiffness, indicated by arrows), the Blade domains undergo a flattening conformational change. This, in turn, induces a rigid-body movement of the intracellular Beam/Anchor domain. The movement of the Beam/Anchor domain effectively pulls apart the Pore Helices, leading to the rapid opening of the central ion pore. This opening facilitates the influx of extracellular calcium ions (Ca^2+^), which serve as a critical second messenger pathway in T cell signaling (exemplified by CAR-T cells).

Piezo1 is the core mediator of mechanical signal transduction in T cells, with CAR-T cells serving as a well-characterized prototype. This unique homotrimeric ion channel can assemble into a large propeller-like structure with a diameter of approximately 24 nm and possesses a distinct dome-shaped topological structure (Ge et al., 2015). When CAR-T cells sense mechanical forces in the microenvironment, such as the kilonewton-scale mechanical forces encountered in a stiff ECM, the structure of Piezo1 undergoes conformational flattening (Zhao et al., 2018). This conformational change not only increases the membrane contact area of the channel, but also drives lever-like movement of the intracellular “beam” domain, ultimately triggering the opening of the central ion pore (Wang et al., 2025). As the primary mechanoreceptor on the surface of CAR-T cells, Piezo1 directly converts physical stimuli in the microenvironment into intracellular biochemical signals. While the channel remains closed in its resting state, once it is opened by direct mechanical stimulation, it promotes the massive influx of Ca^2+^—and Ca^2+^ is precisely the primary downstream second messenger (Figure 2).

### 3.1 Piezo1: a biphasic regulatory hub for T cell fate

T cells are not passive recipients of TME-imposed stress; instead, they are equipped with sophisticated molecular machinery that enables them to sense and transduce physical cues into biological responses (Li et al., 2020; Kankeu Fonkoua et al., 2022). Crucially, the functional consequences of Piezo1 activation are biphasic and context-dependent, dictated by the duration and intensity of the mechanical stimulus (Qu and Zhang, 2025). This duality positions Piezo1 as a central arbiter that can either potentiate T cell-mediated antitumor immunity or drive therapeutic failure. In the context of solid tumor therapy, the adverse effects stemming from chronic Piezo1 activation are particularly prominent, representing a significant challenge for T cell efficacy. At the same time, acute stimulation can enhance antitumor functions (Pang et al., 2024; Abiff et al., 2023).

### 3.2 Negative regulation: Chronic Piezo1 signaling induces exhaustion and impedes migration

In stark contrast to the beneficial effects of acute stimulation, chronic Piezo1 signaling (lasting at least 72 h) has been shown to induce a state of functional exhaustion in T cells, significantly compromising their therapeutic potential (Liu et al., 2018). This phenotypic dysfunction closely mirrors observations within TMEs characterized by elevated stiffness or the presence of abundant TGF-β. Specifically, within the stiff solid TME, Piezo1 is observed to function as an “inhibitor.” Sustained chronic mechanical stress is known to induce excessive activation of Piezo1, leading to intracellular Ca^2+^ overload and thereby triggering a set of detrimental programs (Hope et al., 2022) (Zhang et al., 2021) (Vaeth et al., 2016). On one hand, this activation initiates the YAP/TAZ-dependent pathway, which drives the differentiation of T cells toward an exhausted phenotype (Meng et al., 2020). This phenotype is characterized by the upregulated expression of multiple immunosuppressive checkpoints, such as PD-1, TIM-3, and LAG-3, while the secretion of effector molecules, including granzyme B (GZMB), is inhibited (Guy et al., 2022; Hashimoto et al., 2022; Niu et al., 2019; Hofmann et al., 2024). Mechanistic insights reveal that chronic Piezo1 activation drives sustained Ca^2+^ influx, inhibiting the Hippo signaling pathway and leading to the stabilization of nuclear YAP/TAZ condensates (Lebid et al., 2020). The Hippo pathway, traditionally associated with organ size control, is increasingly recognized as a pivotal mediator of mechanical signaling, with YAP/TAZ serving as downstream effectors that integrate Piezo1-derived Ca^2+^ signals (Meng et al., 2020). Sustained Piezo1 activation elevates HIF-1α via Ca^2+^-calpain signaling (Chen X. et al., 2025), shifting metabolism toward glycolysis and suppressing AMPK-dependent fatty acid oxidation (Leng et al., 2022). Concurrently, the sustained Ca^2+^ influx also activates the CaMKII-CREB-Osr2 signaling axis (Zhang et al., 2021; Opazo et al., 2018; Benito and Barco, 2010). Osr2 has been shown to subsequently recruit HDAC3, mediating epigenetic remodeling that represses the transcription of cytotoxic effector genes such as Interferon gamma (IFN-γ) and Perforin 1 (PRF1), while concurrently upregulating exhaustion-associated markers, thus promoting the establishment of a terminal exhausted state (Zhang et al., 2021). This terminal exhausted phenotype is further characterized by a 40%–50% downregulation of T cell activation markers and suppressed granzyme B secretion (Zhang et al., 2021; Watanabe et al., 2018). While some of these mechanotransduction pathways have been characterized in non-immune cells, emerging evidence suggests a conserved role in T cell dysfunction within the stiff TME.

**FIGURE 3:**
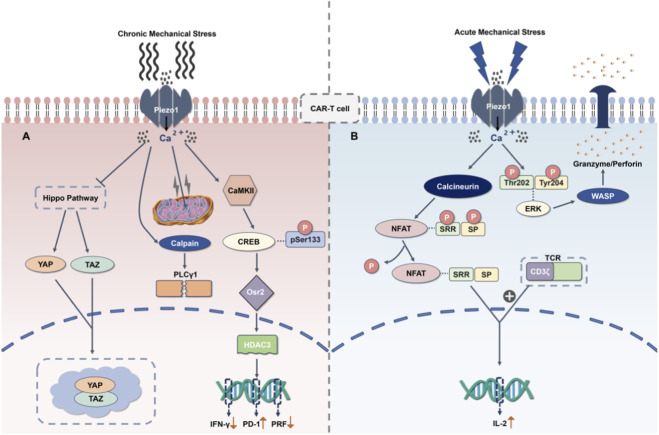
Biphasic Modulation of T Cell Activity by Piezo1 Activation (Exemplified by CAR-T Cells). **(A)** Chronic Piezo1 activation and its inhibitory effects on CAR-T cells. Within the desmoplastic and rigid solid TME, sustained mechanostimulation leads to chronic Piezo1 activation and subsequent intracellular Ca^2+^ overload. This persistent activation consequently triggers inhibitory signaling pathways, notably the Yes-associated protein (YAP)/transcriptional co-activator with PDZ-binding motif (TAZ) axis. Ultimately, this cascade culminates in T cell exhaustion, characterized by the upregulation of exhaustion markers, including Programmed cell death protein 1 (PD-1) and T cell immunoglobulin and mucin-domain containing-3 (TIM-3), a significantly impaired migratory capacity, and diminished cytotoxic function. **(B)** Acute Piezo1 activation and its potentiating effects on CAR-T cells. In contrast, transient mechanical stimuli, such as localized stiffness gradients within the TME or membrane tension fluctuations during immunological synapse formation, induce acute activation of Piezo1. This acute activation, mediated through the Ca^2+^-Nuclear Factor of Activated T cells (NFAT) signaling pathway, significantly enhances the proliferation, cytokine secretion, and antitumor cytotoxicity of T cells (exemplified by CAR-T cells).

Beyond inducing exhaustion, chronic Piezo1 signaling also profoundly impairs cellular motility. Sustained Piezo1 activation has been shown to trigger the excessive polymerization of actin through downstream signaling pathways, such as the ERK-WASP axis, resulting in abnormal rigidity of the cytoskeleton (Roy et al., 2018; Laverty et al., 2022; Jung et al., 2021). This contrasts with Piezo1’s pro-inflammatory role in macrophages (Chen X. et al., 2025) yet aligns with Leng et al. (2022) showing glycolytic-switch impairs AMPK-dependent FAO (Leng et al., 2022). This effect leads T cells to adopt a spread and adherent morphology, completely losing the plasticity required for efficient migration and consequently reducing their migration speed by more than 50% (Figure 3A). Overall, the chronic Piezo1 signaling pathway acts in conjunction with other restrictive factors in the TME. These factors include hypoxic gradients (which induce metabolic exhaustion by stabilizing Hypoxia-Inducible Factor-1 Alpha (HIF-1α)) and immunosuppressive mediators secreted by CAFs, such as TGF-β (Shen et al., 2024; Doedens et al., 2013; Chandra Jena et al., 2021; Zebley et al., 2024). This collaboration results in T cells exhibiting a multifaceted dysfunctional state, which also poses a significant barrier to achieving optimal therapeutic responses in solid tumors.

### 3.3 Positive regulation: Acute Piezo1 signaling facilitates initial activation

Piezo1 is not solely detrimental; under distinctly different stimulation patterns, it shifts to function as an “activator” and exhibits potent pro-functional potential. In sharp contrast to the detrimental effects of chronic signaling, transient mechanical cues and acute Piezo1 activation (pulse duration <2 h; peak [Ca^2+^] ≤ 800 nM) are crucial for the initial activation and cytotoxic capacity of T cells (Hope et al., 2022). Under acute stimulation, for instance, during the initial contact between T cells and tumors, transient, low-intensity mechanical stimulation triggers a transient Ca^2+^ influx. This acute Piezo1-mediated Ca^2+^ influx activates calcineurin, which dephosphorylates and promotes nuclear translocation of the transcription factor Nuclear Factor of Activated T cells (NFAT) (Ren et al., 2023; Kalluri, 2016; Tomida et al., 2003). Activated NFAT then translocates into the nucleus, where it preferentially activates the classical NFAT signaling pathway. In the nucleus, NFAT synergizes with CAR-derived signals (specifically from the CD3ζ domain) to promote cell proliferation and upregulate the transcription of interleukin-2 (IL-2), along with the secretion of effector cytokines, including interferon-γ (IFN-γ) and GZMB, thereby significantly boosting CAR-T cells’ cytotoxicity. Interestingly, this acute Ca^2+^ flux simultaneously enhances OXPHOS efficiency (Leng et al., 2022), providing immediate ATP for cytotoxicity. This process is considered crucial for stabilizing immune synapses and achieving efficient target cell killing, highlighting a potential therapeutic opportunity. This Ca^2+^ signal is also fundamental for cytoskeletal remodeling (via Ca^2+^-dependent phosphorylation of ERK and WASP) and the stabilization of the immunological synapse, both of which are critical for efficient target cell lysis and initial tumor elimination (Mao et al., 2023; Babich and Burkhardt, 2013; Chao et al., 2025). The pro-functional effects starkly contrast with Piezo1-driven glycolytic metabolism during chronic stimulation (Chen X. et al., 2025), indicating timing-dependent metabolic rewiring. Notably, such acute Piezo1 activation may occur not only during initial T cell-tumor contact but also in response to dynamic mechanical changes within the TME (Aguilar et al., 2017). This transient signaling profile starkly contrasts with chronic Piezo1 activation (duration >72 h; sustained [Ca^2+^] > 1.2 µM), which induces mitochondrial Ca^2+^ overload and calpain-mediated cleavage of (Phospholipase C gamma 1)PLCγ1, ultimately impairing CAR signaling and driving cellular exhaustion (Zhu et al., 2022; Sun et al., 2025). This functional dichotomy highlights the context-dependent role of Piezo1 in determining the fate and function of T cells within the TME (Figure 3B).

Collectively, these findings underscore the pivotal role of the Piezo1 signaling system as a “molecular switch” that fundamentally dictates the functional trajectory of T cells (exemplified by CAR-T cells) (Wu and Ye, 2025). It establishes a direct mechanistic link between the biophysical properties of the TME and the antitumor potency of T cells (exemplified by CAR-T cells), finely tuning their functional equilibrium between robust cytolytic activity and a state of exhaustion coupled with adhesion (Luo et al., 2022). Therefore, Piezo1 stands out as a preeminent therapeutic target for mitigating the physical impediments of solid tumors, unequivocally pointing the way for subsequent research.

### 3.4 The indirect regulatory effects of Piezo1 on the TME

The profound impact of Piezo1 on the fate of T cells is increasingly recognized to extend beyond a solely cell-intrinsic phenomenon (Hong et al., 2023; Good et al., 2021). A comprehensive understanding of its function requires an appreciation of its role within the entire TME ecosystem, where Piezo1 activity in stromal and cancer cells is known to actively shape the biomechanical and immunological landscape that T cells must navigate (Canella et al., 2023). Notably, beyond Piezo1, other mechanosensitive channels also significantly influence immune cell function in the TME. TRPM7 mediates Ca^2+^ influx in T cells under shear stress, thereby regulating cell migration and cytokine production (Starostina et al., 2021). TRPV4 responds to changes in extracellular matrix stiffness and osmolarity, affecting the infiltration efficiency of T cells in solid tumors (Scheraga et al., 2020). Although Piezo2 is primarily expressed in sensory neurons, it regulates neuro-immune crosstalk and indirectly modulates the biomechanical properties of the TME (Nagase et al., 2024; Nilius and Honoré, 2012). These channels collectively form an integrated mechanosensory network that deciphers diverse mechanical cues in the TME, synergizing with Piezo1 to fine-tune immune cell behavior. This section provides an overview of Piezo1 functions, its activating factors, and its indirect effects on T cells across various TME cell types, such as CAFs and macrophages (Table 2). This broader network of mechanotransduction is consequently implicated in establishing a vicious cycle of feedback. Here, mechanical stress not only directly impairs T cells but also instructs other cell types to reinforce the immunosuppressive barrier, thereby collectively presenting a significant challenge to the efficacy of T cells in solid tumors and highlighting a critical area for strategic intervention (Zhu et al., 2025). Targeting the integrated mechanosensory network composed of Piezo1, TRPM7, TRPV4, and Piezo2 may help to overcome T cell resistance through multiple pathways. TRPM7 inhibition attenuates Ca^2+^ overload and maintains T cell migratory capacity in dense matrices (Wu W. et al., 2023). Blocking TRPV4 prevents stiffness-induced actin hyper-polymerization, thereby enhancing immune cell infiltration (Goswami et al., 2021). Suppressing Piezo2-dependent sensory neuron signaling diminishes CAF activation and ECM stiffening (Woo et al., 2015).

**TABLE 2:** Differential roles of Piezo1 in TME cell types and its indirect impact on T Cell therapeutic efficacy.

Cell type	Piezo1 activator	Key Piezo1-Mediated effects	Indirect impact on T cells	Therapeutic strategy	Validation method	References
CAFs	ECM stiffness, Mechanical strain	Pro-fibrotic and immunosuppressive (α-SMA↑, Collagen I↑, TGF-β↑)	Physical barrier hinders infiltration; TGF-β suppresses activity	TGF-β inhibitors	*In vitro*: Co-culture, Transwell assays	(Wei et al., 2019; Zhu et al., 2023)
				LOXL2 inhibitors	*In vivo*: Piezo1-KO CAF tumor models	
TAMs	Hypoxia, Mechanical pressure	Promotes M2 polarization (IL-10↑, CD206↑); MMP secretion↑	Induces exhaustion (PD-1↑); Disrupts migration paths	Piezo1 antagonists (e.g., Gsmtx4, an inhibitor)	*Ex vivo*: Flow cytometry of primary TAMs	(Solis et al., 2019)
				TAM polarization modulators (e.g., CSF1R inhibitors)	*In vivo*: Systemic Gsmtx4 in tumor models	

α-SMA, α-Smooth Muscle Actin; CAFs, Cancer-Associated Fibroblasts; CSF1R, Colony-Stimulating Factor 1 Receptor; IL-10, Interleukin-10; CD206 Cluster of Differentiation 206; KO, knockout; LOXL2, Lysyl Oxidase-Like 2; MMP, matrix metalloproteinase; TAMs, Tumor-Associated Macrophages.

**FIGURE 4:**
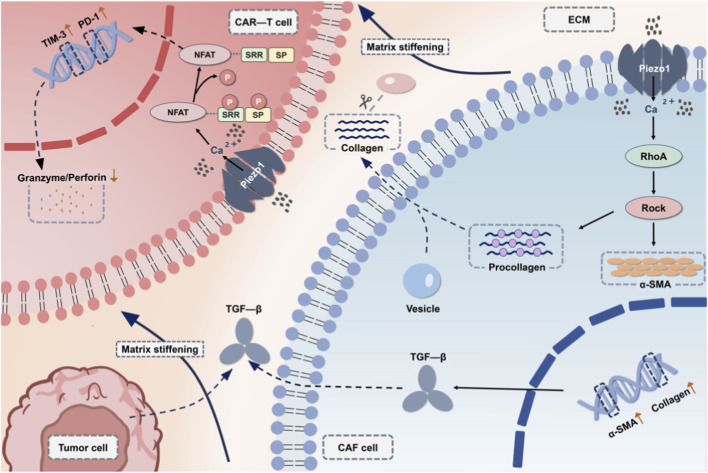
Piezo1-mediated positive feedback loop in cancer-associated fibroblasts (CAFs) driving T cell-targeted physical barrier formation in the TME (exemplified by CAR-T cells).

CAFs are universally acknowledged as the indispensable orchestrators of the desmoplastic stroma (Kalluri, 2016). Mounting evidence now highlights Piezo1 as a pivotal regulator of CAF activation, thereby profoundly influencing their phenotype and functional contributions (Park et al., 2023). Within a stiffening TME, increased mechanical strain on CAFs has been shown to activate their endogenous Piezo1 channels (Vasileva et al., 2021). This activation triggers Ca^2+^-dependent signaling cascades, such as the Ras Homolog Gene Family Member A/Rho-Associated Protein Kinase (RhoA/ROCK) pathway, which promotes the expression of alpha-smooth muscle actin (α-SMA) and drives their differentiation into a myofibroblast-like, pro-fibrotic phenotype. These activated CAFs, in turn, accelerate the deposition and crosslinking of ECM proteins, particularly type I collagen, thereby further increasing matrix stiffness (Zhang et al., 2025). Specifically, the increased mechanical stress (stiffness) within the TME is sensed by Piezo1 channels on the surface of CAFs, leading to Ca^2+^influx. The elevated intracellular Ca^2+^ concentration subsequently activates downstream signaling pathways, including NFAT and RhoA/ROCK, which are understood to be key regulators of cytoskeletal rearrangement, gene transcription, and the synthesis and secretion of ECM components such as collagen and fibronectin (Tang et al., 2018). Ultimately, this process culminates in the formation of a denser and stiffer ECM barrier, which effectively impedes T cells’ (exemplified by CAR-T cells) infiltration into the tumor core region and drives the chronic mechanical stress that leads to T cells’ exhaustion, thus constituting a significant bottleneck in therapeutic delivery (Camacho-Gomez et al., 2024) (Figure 4). Furthermore, Piezo1-activated CAFs are also known to enhance the secretion of immunosuppressive cytokines, including TGF-β (Swain et al., 2022).

Piezo1’s function is not an isolated phenomenon; instead, it is intricately integrated within a broader cellular mechanosensory apparatus that includes other ion channels, such as TRPV4, integrins, and focal adhesion complexes (Yu and Liao, 2021; Qiu et al., 2019). The signals originating from Piezo1-mediated Ca^2+^ influx are understood to be integrated with these other pathways to orchestrate a cohesive cellular response (Saotome et al., 2018). For example, the membrane depolarization caused by Piezo1 can modulate the activity of voltage-gated channels. At the same time, Ca^2+^ signals can influence integrin clustering and focal adhesion dynamics, thereby affecting cell adhesion and migration (De Felice and Alaimo, 2020). A comprehensive understanding of this crosstalk is therefore essential for predicting the full consequences and potential opportunities of therapeutic modulation of Piezo1 (Neophytou et al., 2025; Xu et al., 2025). The pleiotropic effects of Piezo1, coupled with the spatial heterogeneity of mechanical cues within the TME, collectively pose significant challenges that necessitate a holistic view of the entire mechanosensory network (Lai et al., 2022; Pang et al., 2024). Future research, particularly leveraging emerging technologies such as spatial transcriptomics and advanced *in vivo* biosensors, is anticipated to be crucial in dissecting this inherent complexity and identifying more precise directions and specific targets for therapeutic intervention (Park et al., 2023; Jin et al., 2024).

## 4 Combined therapeutic strategies targeting the TME- Piezo1 axis

### 4.1 Remodeling the tumor mechanical microenvironment

This extrinsic approach focuses on alleviating the biophysical stiffness of the TME, thereby potentially reducing the chronic Piezo1 stimulation that drives T cell exhaustion and enhancing physical accessibility for infiltration (Zheng et al., 2024). These physical and mechanical barriers are understood to be primarily composed of abnormally increased ECM stiffness, IFP, and solid stress, which collectively form a complex network that impairs T cells’ function.

**FIGURE 5:**
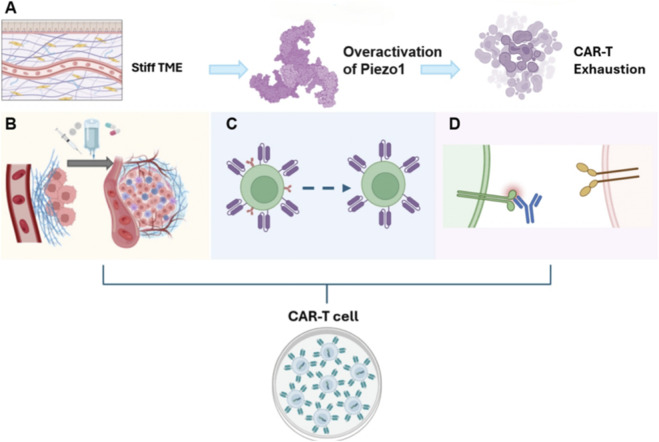
Therapeutic interventions targeting the TME- Piezo1 axis to overcome mechanical barriers and augment T cells’ efficacy in solid tumors (exemplified by CAR-T cells). **(A)** Mechanism of Dysfunction: In desmoplastic solid tumors, characterized by elevated matrix stiffness, CAR-T cells (as a representative of therapeutic T cells) experience sustained and aberrant activation of their Piezo1 channels. This leads to detrimental intracellular Ca^2+^ overload, which critically drives the exhaustion of these T cells. Such exhaustion is characterized by the upregulation of inhibitory checkpoints, including Programmed cell death protein 1 (PD-1) and Lymphocyte-activation gene 3 (LAG-3), reduced cytotoxic function, and impaired tumor infiltration capabilities. **(B)** Microenvironmental Remodeling: This extrinsic strategy involves interventions designed to alleviate the physical stiffness of the TME. Approaches include the use of ECM-degrading enzymes, vascular normalization drugs, or targeted therapeutics engineered to mitigate solid stress or degrade the ECM. By reducing TME stiffness, this strategy aims to diminish chronic Piezo1 mechanostimulation at its origin, creating a more permissive, ‘softer’ milieu that facilitates the restoration of effector functions in T cells (exemplified by CAR-T cells in this figure). **(C)** Direct Piezo1 Modulation: This pharmacological strategy involves direct intervention on T cells (exemplified by CAR-T cells in this figure) using specific Piezo1 antagonists. These antagonists inhibit Piezo1 channel opening, effectively abrogating aberrant Ca^2+^ signaling cascades even within a persistently rigid TME. This direct modulation safeguards these T cells from mechanosensitive exhaustion. **(D)** Genetic Engineering of CAR-T cells: This intrinsic approach leverages genetic engineering tools, such as CRISPR-Cas9, to modify CAR-T cells (a prototype of engineered therapeutic T cells). Downregulating or abrogating the expression of the Piezo1 gene renders these T cells mechanistically ‘insensitive’ to inhibitory signals originating from the rigid TME. This fundamental genetic modification is designed to augment their persistence and therapeutic efficacy within desmoplastic tumors.

One primary strategic direction involves targeting the function of CAFs, given their established role as the principal source of ECM deposition. Pharmacological agents targeting key fibrotic pathways, such as inhibitors of TGF-β (e.g., Fresolimumab) or Lysyl Oxidase Like 2 (LOXL2), which is crucial for collagen crosslinking, represent promising avenues to decrease matrix stiffness (Yamazaki et al., 2022; Formenti et al., 2018; Cheng et al., 2023). By disrupting the positive feedback loop where mechanical strain promotes CAF activation and further fibrosis (Wang et al., 2023), these agents have the potential to create a more permissive environment for T cell activity (Figure 5A). Clinical trials investigating these agents in combination with immunotherapies are currently underway, and their application alongside CAR-T therapy (a key form of T cell immunotherapy) represents a logical next strategic step (Liu et al., 2021).

A more direct approach involves the enzymatic degradation of the ECM itself (Klabukov et al., 2025; Mayorca-Guiliani et al., 2025). The co-administration of matrix-degrading enzymes, such as collagenase or hyaluronidase, can effectively degrade the dense matrix, thereby increasing the interstitial space and enhancing permeability for T cell infiltration (Zhao et al., 2025). (Figure 5B) While the systemic delivery of such enzymes presents inherent challenges related to toxicity and immunogenicity, novel platforms are being actively explored to achieve localized efficacy (Chen A. X. Y. et al., 2025). These innovative strategies include engineering CAR-T cells to secrete these enzymes directly at the tumor site or employing nanoparticle-based systems that release their enzymatic cargo only in response to tumor-specific triggers, such as low pH or specific enzyme activity (Yin et al., 2025).

Furthermore, drugs initially developed for other indications may offer additional prospects through beneficial matrix-remodeling effects. For example, the angiotensin II receptor blocker Losartan has been observed to inhibit TGF-β signaling in CAFs, leading to reduced collagen production and improved chemotherapy delivery in pancreatic cancer models (Mei et al., 2024). Such repurposing strategies present an expedited path to clinical translation and could readily be combined with T cell infusions, representing a significant opportunity for rapid therapeutic advancement (Schuster et al., 2017).

### 4.2 Direct pharmacological modulation of Piezo1

Directly targeting the Piezo1 channel with small-molecule modulators represents a promising yet intricate strategy, primarily due to its established biphasic role in the function of T cells (Luu et al., 2025). The overarching objective involves selectively inhibiting chronic, exhaustion-inducing signaling pathways while preserving, or even enhancing, acute, activation-promoting signals (Figure 5C). A significant impediment to this approach arises from the systemic application of such modulators. Systemic administration of a Piezo1 antagonist (e.g., Gsmtx4) carries the inherent risk of blocking the initial mechanostimulation critical for immunological synapse formation and T cell activation (Coste et al., 2010). Conversely, the systemic use of a Piezo1 agonist, such as the Piezo1-selective agonist Yoda1 (a well-characterized synthetic small-molecule tool compound that directly binds to Piezo1 channels to promote gating and Ca^2+^ influx) could induce widespread chronic activation, potentially exacerbating T cell exhaustion and eliciting significant off-target effects in other mechanosensitive tissues, including endothelial cells and erythrocytes (Kuck et al., 2024; Xu et al., 2022). This observation underscores a critical limitation of systemic Piezo1 modulation: its pleiotropic expression across diverse tissues (Niu et al., 2025). For instance, Piezo1 is essential for vascular development and erythrocyte volume regulation; therefore, its inhibition could potentially lead to unforeseen cardiovascular or hematological complications, thereby necessitating the development of highly localized delivery strategies. As consistently highlighted in existing reviews on Piezo channels, their ubiquitous expression and pleiotropic physiological functions mandate highly targeted approaches, thus necessitating locally restricted modulation strategies. For example, novel approaches include nanoparticle-encapsulated Piezo1 antagonists engineered for tumor-selective release via enzyme-responsive biodegradation (Raju et al., 2022) and photo-switchable Piezo1 agonists (e.g., optogenetically controlled Yoda1 derivatives) activated solely by focused near-infrared light within tumors (Yu et al., 2022). Such spatial precision could uncouple beneficial acute signaling from detrimental chronic activation while minimizing off-target effects in vascular and erythroid systems (Wang et al., 2025; Lüchtefeld et al., 2024).

Addressing this challenge requires precise, tumor-localized drug delivery. Advanced drug delivery systems offer the potential to provide the requisite spatiotemporal control. Complementary metabolic interventions (e.g., HIF-1α inhibitor PX-478 or AMPK activator Metformin) may reverse chronic Piezo1-driven dysfunction without direct channel manipulation (Chen X. et al., 2025; Leng et al., 2022). For example, CAR-T cells could be engineered with nanoparticles encapsulating a Piezo1 agonist, such as Yoda1. These nanoparticles could be designed to release their payload specifically upon stable synapse formation with a tumor cell, thereby delivering a potent yet transient pulse of Piezo1 activation to maximize initial cytotoxicity (Khairullina et al., 2025). A forward-looking avenue involves the development of photoswitchable Piezo1 modulators, which could be systemically administered in an inactive form and subsequently activated by focused light exclusively within the tumor mass, offering unparalleled spatiotemporal precision (Antosik et al., 2025).

### 4.3 Intrinsic engineering of T cells for mechanical resilience

This cell-intrinsic approach presents a compelling avenue to reprogram T cells (with CAR-T cells as a prototype) to become refractory to, or functionally reinterpret, the suppressive mechanical cues of the TME. One established method leverages gene-editing technologies, such as CRISPR-Cas9, to delete or modify the Piezo1 gene in CAR-T cells prior to infusion (Figure 5D).

Piezo1-knockout CAR-T cells are theoretically rendered unresponsive to the chronic mechanical stress of the TME, effectively uncoupling the stiff environment from the intracellular exhaustion program (Pang et al., 2024). Preclinical studies have demonstrated that this strategy can preserve the functionality of T cells in stiff 3D hydrogels and enhance tumor control *in vivo* (Revilla et al., 2025; Leonardi et al., 2025). A more sophisticated paradigm involves engineering next-generation CAR constructs that fundamentally repurpose the mechanical stress signal from an inhibitory to an activating input. These advanced constructs could incorporate novel mechanically regulated signaling domains. For example, a synthetic domain could be designed to specifically bind to YAP/TAZ only when they translocate to the nucleus under high mechanical stress (Duan et al., 2025; Wang et al., 2017). This binding could then trigger a potent co-stimulatory signal, originating from a domain such as CD137 or CD28, rather than initiating an exhaustion program (Yang et al., 2025). Such an innovative design holds substantial promise for creating engineered T cells (e.g., CAR-T cells) that remain quiescent in compliant, healthy tissues but become fully activated upon encountering the high-stiffness signature of a solid tumor, thereby maximizing on-target potency while minimizing off-tumor toxicity (Lv et al., 2025; Adu-Berchie et al., 2023).

Translating foundational research into viable clinical applications for TME- Piezo1 modulation is confronted by several significant bottlenecks. Firstly, spatial mechanical heterogeneity poses a substantial obstacle, as steep stiffness gradients exist between rigid hypoxic cores and more compliant vascularized peripheries, compromising uniform therapeutic efficacy. Resolving this complexity demands the development of spatially adaptable interventions capable of achieving region-specific activity modulation (Mazloomi et al., 2025; Fu et al., 2025; Holt et al., 2021). Secondly, cell-type-specific pleiotropy further complicates targeted approaches, wherein Piezo1 activation can simultaneously impair T cells function while promoting protumorigenic processes in stromal components, including CAFs and macrophages. Rigorous evaluation of the long-term safety profiles for Piezo1-targeting approaches, particularly regarding their impact on systemic immune homeostasis and physiological mechanotransduction, remains unequivocally imperative before widespread clinical adoption (Du et al., 2025; Song et al., 2019).

Therapies targeting Piezo mechanosensitive ion channels are currently in a critical stage of translation from fundamental discovery to clinical application. As core molecules of mechanotransduction, Piezo channels were first molecularly identified in 2010 (Coste et al., 2012). Their role in regulating various pathophysiological processes has been widely confirmed, and relevant research has been further recognized by the 2021 Nobel Prize in Physiology or Medicine. However, an undeniable fact is that the development of Piezo channels as drug targets remains a vast but relatively underdeveloped frontier. Currently, no drugs targeting Piezo1/2 have entered Phase I-III clinical trials worldwide, and drug development in this area remains a frontier with relatively slow progress (Wu et al., 2017). There are multiple underlying reasons behind this translational lag: First, the relatively late discovery of Piezo as a therapeutic target has led to a prolonged exploration cycle. Its complete biological function landscape remains incomplete, and its roles in basic physiological processes such as touch sensation and blood pressure regulation were only gradually clarified after 2010 (Geng et al., 2017). Second, the widespread systemic distribution of Piezo channels renders tissue-specific targeting highly challenging, making them prone to inducing off-target effects. Finally, the inherent difficulty in drug development cannot be overlooked. Designing small molecules or biologics with high selectivity, high affinity, and favorable druggability against such large, trimeric membrane proteins is inherently a formidable technical challenge.

Despite numerous obstacles, research and development activities continue to advance in several key areas, demonstrating a clear translational pathway. In the field of genetic diseases, breakthroughs have been achieved in addressing hereditary stomatocytosis, which is caused by gain-of-function mutations in Piezo1 (Demolombe et al., 2013). In fields such as chronic pain, cardiovascular diseases, and osteoporosis, preclinical studies targeting Piezo channels are also being conducted in an orderly manner. They are expected to provide novel therapeutic solutions for these diseases, which have significant unmet medical needs (Kim et al., 2022). Focusing on the core of this study, targeting the TME- Piezo1 axis in the field of tumor immunotherapy to enhance the efficacy of cellular immunotherapy is also a cutting-edge application direction in this field. Numerous preclinical studies have demonstrated that Piezo1 influences cytotoxic efficacy by regulating T cell traction force, and that desensitizing the mechanical sensing ability of T cells (including CAR-T cells) via small-molecule inhibitors or genetic engineering approaches such as CRISPR to knockout Piezo1 is an effective strategy for overcoming the physical barriers of solid tumors and reversing T cell exhaustion (Pang et al., 2024). Although they have not yet entered clinical trials, these findings provide critical theoretical support for overcoming the solid tumor bottleneck in cellular therapy. In summary, the development of “mechano-drugs” targeting Piezo channels represents a paradigm shift in drug development. With the in-depth analysis of its structural pharmacology (Papavassiliou et al., 2023; Tang et al., 2022), combined with advanced drug design and genetic engineering technologies, we have sound reasons to believe that the first batch of innovative therapies targeting Piezo channels is expected to enter clinical practice within the next decade, bringing revolutionary therapeutic breakthroughs to a range of refractory diseases, including genetic diseases and tumors.

## 5 Conclusion

This review highlights that targeting Piezo1, a pivotal mechanosensitive ion channel mediating the physical barrier of the solid TME, represents a highly promising, multifaceted strategy to significantly bolster the efficacy of T cell-based immunotherapies (including CAR-T cell therapy) against solid tumors. Nevertheless, the clinical translation of this approach encounters formidable challenges, including the necessity for a more comprehensive elucidation of its intricate cell- and environment-dependent signaling mechanisms, the achievement of precise cellular selectivity to mitigate off-target effects, and establishing standardized biomechanical biomarkers for optimal patient stratification and efficacy prediction. Advances in these areas will substantially expand the applicability of T cell-based immunotherapies, particularly CAR-T cell therapies, thereby benefiting a larger cohort of patients with solid tumors and improving clinical outcomes.

## Glossary

CAR-T: Chimeric Antigen Receptor T cell
TME: Tumor microenvironment
Piezo1: Piezo-type mechanosensitive ion channel component 1
YAP/TAZ: Yes-associated protein/Transcriptional co-activator with PDZ-binding motif
IFP: Interstitial fluid pressure
ECM: Extracellular matrix
NK: Natural Killer
TAMs: Tumor-associated macrophages
MDSCs: Myeloid-derived suppressor cells
DCs: Dendritic cells
Treg: Regulatory T
CAFs: Cancer-associated fibroblasts
PDAC: Pancreatic ductal adenocarcinoma
TNBC: Triple-negative breast cancer
TGF-β: Transforming growth factor-beta
IL-10: Interleukin-10
PD-1: Programmed death-1
LAG-3: Lymphocyte-activation gene 3
TRPV4: Transient receptor potential vanilloid subtype 4
Piezo2: Piezo-type mechanosensitive ion channel component 2
ERK: Extracellular signal-regulated kinase
WASP: Wiskott-Aldrich syndrome protein
**Ca** ^**2+**^: Calcium ion
PRF1: Perforin-1
CEUS: Contrast-enhanced ultrasound
CT: Computed tomography
MRE: Magnetic resonance elastography
GZMB: Granzyme B
TIM-3: T cell immunoglobulin and mucin-domain containing-3
CaMKII: Calcium/calmodulin-dependent protein kinase II
CREB: cAMP response element-binding protein
Osr2: Odd-skipped related 2
HDAC3: Histone deacetylase 3
HIF-1α: Hypoxia-inducible factor-1 alpha
CD3ζ: CD3 zeta chain
NFAT: Nuclear factor of activated T cells
IL-2: Interleukin-2
IFN-γ: Interferon-gamma
PLCγ1: Phospholipase C gamma 1
α-SMA: α-smooth muscle actin
RhoA/ROCK: Ras homolog gene family member A/Rho-associated protein kinase
MMP: Matrix metalloproteinase
CD206: Cluster of differentiation 206
CSF1R: Colony-stimulating factor 1 receptor
Gsmtx4: Giant squid metallocarboxypeptidase inhibitor 4
KO: Knockout
CD8: Cluster of differentiation 8
CD11b: Integrin alpha M
CD4: Cluster of differentiation 4
CSPG4: Chondroitin sulfate proteoglycan 4
CAR-macrophages: Chimeric antigen receptor macrophages
BLIMP1: B-lymphocyte-induced maturation protein 1
NR4A3: Nuclear receptor subfamily 4 group A member 3
NAMPT: Nicotinamide phosphoribosyltransferase
cGAS: Cyclic GMP-AMP synthase
STING: Stimulator of interferon genes
LOXL2: Lysyl oxidase-like 2
JNK: c-Jun N-terminal kinase
CRISPR-Cas9: Clustered regularly interspaced short palindromic repeats-associated protein 9
CD137: Tumor necrosis factor receptor superfamily member 9
CD28: Cluster of differentiation 28
TLR7/8: Toll-like receptor 7/8
PPARγ: Peroxisome proliferator-activated receptor gamma
NECTIN4: Nectin cell adhesion molecule 4
ACT: adoptive T cell therapies

## References

[B1] Abiff M. Alshebremi M. Bonner M. Myers J. T. Kim B. G. Tomchuck S. L. (2023). Piezo1 facilitates optimal T cell activation during tumor challenge. Oncoimmunology 12, 2281179. 10.1080/2162402X.2023.2281179 38126029 PMC10732680

[B2] Acerbi I. Cassereau L. Dean I. Shi Q. Au A. Park C. (2015). Human breast cancer invasion and aggression correlates with ECM stiffening and immune cell infiltration. Integr. Biol. (Camb) 7, 1120–1134. 10.1039/c5ib00040h 25959051 PMC4593730

[B3] Adu-Berchie K. Liu Y. Zhang D. K. Y. Freedman B. R. Brockman J. M. Vining K. H. (2023). Generation of functionally distinct T cell populations by altering the viscoelasticity of their extracellular matrix. Nat. Biomed. Eng. 7, 1374–1391. 10.1038/s41551-023-01052-y 37365267 PMC10749992

[B4] Aguilar A. V. Benson B. Rathkey J. Correa L. Li L. Myers J. (2017). Live-cell visualization of gasdermin D-driven pyroptotic cell death. J. Biol. Chem. 292 (35), 14649–14658. 10.1074/jbc.M117.797217 28726636 PMC5582855

[B5] Ai J. Li H. Zhang M. Liu J. Liu L. Sun C. (2026). Mechanical microenvironment in tumor immune evasion: bidirectional regulation between matrix stiffness and immune cells and its therapeutic implications. Int. J. Biol. Sci. 22, 280–307. 10.7150/ijbs.121356 41362727 PMC12681854

[B6] Albelda S. M. (2024). CAR T cell therapy for patients with solid tumours: key lessons to learn and unlearn. Nat. Rev. Clin. Oncol. 21, 47–66. 10.1038/s41571-023-00832-4 37904019

[B7] Angeli S. Neophytou C. Kalli M. Stylianopoulos T. Mpekris F. (2025). The mechanopathology of the tumor microenvironment: detection techniques, molecular mechanisms and therapeutic opportunities. Front. Cell Dev. Biol. 13, 1564626. 10.3389/fcell.2025.1564626 40171226 PMC11958720

[B127] Anonymous (2024). Targeting the Piezo1 pathway boosts T cell antitumour cytotoxicity. Nat. Biomed. Eng., 8 (9), 1071–1072. 10.1038/s41551-024-01189-4 38538845

[B8] Antosik P. Szachniewicz M. Baran M. Bonowicz K. Jerka D. Motylewska E. (2025). Piezo-type mechanosensitive ion channel component 1 (Piezo1) as a potential prognostic marker in renal clear cell carcinoma. Int. J. Mol. Sci. 26, 6598. 10.3390/ijms26146598 40724850 PMC12295294

[B9] Babich A. Burkhardt J. K. (2013). Coordinate control of cytoskeletal remodeling and calcium mobilization during T cell activation. Immunol. Rev. 256, 80–94. 10.1111/imr.12123 24117814 PMC3824381

[B10] Barbazan J. Pérez-González C. Gómez-González M. Dedenon M. Richon S. Latorre E. (2023). Cancer-associated fibroblasts actively compress cancer cells and modulate mechanotransduction. Nat. Commun. 14, 6966. 10.1038/s41467-023-42382-4 37907483 PMC10618488

[B11] Benito E. Barco A. (2010). CREB's control of intrinsic and synaptic plasticity: implications for CREB-Dependent memory models. Trends Neurosci. 33 (5), 230–240. 10.1016/j.tins.2010.02.001 20223527

[B12] Biray Avci C. Goker Bagca B. Nikanfar M. Takanlou L. S. Takanlou M. S. Nourazarian A. (2024). Tumor microenvironment and cancer metastasis: molecular mechanisms and therapeutic implications. Front. Pharmacol. 15, 1442888. 10.3389/fphar.2024.1442888 39600368 PMC11588459

[B13] Bonner M. Askew D. Sathish Kumar V. Tomchuck S. L. Eid S. Abiff M. (2025). Piezo1 deletion enhances cross-priming of CD8+ T cells by tumor-infiltrating CD11b+ dendritic cells. J. Immunother. Cancer 13, e011815. 10.1136/jitc-2025-011815 40550569 PMC12186042

[B14] Camacho-Gomez D. Movilla N. Borau C. Martin A. Oñate Salafranca C. Pardo J. (2024). An agent-based method to estimate 3D cell migration trajectories from 2D measurements: quantifying and comparing T vs CAR-T 3D cell migration. Comput. Methods Programs Biomed. 255, 108331. 10.1016/j.cmpb.2024.108331 39068872

[B15] Canella A. Nazzaro M. Rajendran S. Schmitt C. Haffey A. Nigita G. (2023). Genetically modified IL2 bone-marrow-derived myeloid cells reprogram the glioma immunosuppressive tumor microenvironment. Cell Rep. 42, 112891. 10.1016/j.celrep.2023.112891 37516967

[B16] Chandra Jena B. Sarkar S. Rout L. Mandal M. (2021). The transformation of cancer-associated fibroblasts: current perspectives on the role of TGF-β in CAF mediated tumor progression and therapeutic resistance. Cancer Lett. 520, 222–232. 10.1016/j.canlet.2021.08.002 34363903

[B17] Chao Z. Mei Q. Yang C. Luo J. Liu P. Peng H. (2025). Immunological synapse: structures, molecular mechanisms and therapeutic implications in disease. Signal Transduct. Target Ther. 10, 254. 10.1038/s41392-025-02332-6 40784895 PMC12336355

[B18] Chen S. Saeed A. F. U. H. Liu Q. Jiang Q. Xu H. Xiao G. G. (2023). Macrophages in immunoregulation and therapeutics. Signal Transduct. Target. Ther. 8, 207. 10.1038/s41392-023-01452-1 37211559 PMC10200802

[B19] Chen X. Jiang J. He B. Luo S. Tan Q. Yao Y. (2025a). Piezo1 aggravates ischemia/reperfusion-induced acute kidney injury by Ca2+-dependent calpain/HIF-1α/Notch signaling. Ren. Fail. 47, 2447801. 10.1080/0886022X.2024.2447801 39780511 PMC11721879

[B20] Chen A. X. Y. Yap K. M. Kim J. S. Sek K. Huang Y.-K. Dunbar P. A. (2025b). Rewiring endogenous genes in CAR T cells for tumour-restricted payload delivery. Nature 644, 241–251. 10.1038/s41586-025-09212-7 40604285 PMC12328239

[B21] Cheng F. Yang F. Wang Y. Zhou J. Qian H. Yan Y. (2023). Mesenchymal stem cell-derived exosomal miR-27b-3p alleviates liver fibrosis *via* downregulating YAP/LOXL2 pathway. J. Nanobiotechnology 21, 195. 10.1186/s12951-023-01942-y 37328872 PMC10273609

[B22] Chow A. Perica K. Klebanoff C. A. Wolchok J. D. (2022). Clinical implications of T cell exhaustion for cancer immunotherapy. Nat. Rev. Clin. Oncol. 19, 775–790. 10.1038/s41571-022-00689-z 36216928 PMC10984554

[B23] Corbet C. Feron O. (2017). Tumour acidosis: from the passenger to the driver's seat. Nat. Rev. Cancer 17, 577–593. 10.1038/nrc.2017.77 28912578

[B24] Coste B. Mathur J. Schmidt M. Earley T. J. Ranade S. Petrus M. J. (2010). Piezo1 and Piezo2 are essential components of distinct mechanically activated cation channels. Science 330, 55–60. 10.1126/science.1193270 20813920 PMC3062430

[B25] Coste B. Xiao B. Santos J. S. Syeda R. Grandl J. Spencer K. S. (2012). Piezo proteins are pore-forming subunits of mechanically activated channels. Nature 483, 176–181. 10.1038/nature10812 22343900 PMC3297710

[B26] Davenport A. J. Cross R. S. Watson K. A. Liao Y. Shi W. Prince H. M. (2018). Chimeric antigen receptor T cells form nonclassical and potent immune synapses driving rapid cytotoxicity. Proc. Natl. Acad. Sci. U. S. A. 115, E2068–e2076. 10.1073/pnas.1716266115 29440406 PMC5834689

[B27] Demolombe S. Duprat F. Honoré E. Patel A. (2013). Slower Piezo1 inactivation in dehydrated hereditary stomatocytosis (xerocytosis). Biophys. J. 105, 833–834. 10.1016/j.bpj.2013.07.018 23972832 PMC3752110

[B28] Deng B. Zhao Z. Kong W. m. Han C. Shen X. Zhou C. (2022). Biological role of matrix stiffness in tumor growth and treatment. J. Transl. Med. 20, 540. 10.1186/s12967-022-03768-y 36419159 PMC9682678

[B29] Discher D. E. Janmey P. Wang Y.-l. (2005). Tissue cells feel and respond to the stiffness of their substrate. Science 310, 1139–1143. 10.1126/science.1116995 16293750

[B30] Doedens A. L. Phan A. T. Stradner M. H. Fujimoto J. K. Nguyen J. V. Yang E. (2013). Hypoxia-inducible factors enhance the effector responses of CD8(+) T cells to persistent antigen. Nat. Immunol. 14, 1173–1182. 10.1038/ni.2714 24076634 PMC3977965

[B31] Du B. Qin J. Lin B. Zhang J. Li D. Liu M. (2025). CAR-T therapy in solid tumors. Cancer Cell 43, 665–679. 10.1016/j.ccell.2025.03.019 40233718

[B32] Duan J. Li H. Zhang J. Xu H. Gao J. Cai M. (2025). Piezo1 affects cell growth and migration *via* microfilament-mediated YAP trans-Latitudinal regulation. Anal. Chem. 97, 147–156. 10.1021/acs.analchem.4c03420 39729436

[B33] Eyquem J. Mansilla-Soto J. Giavridis T. van der Stegen S. J. Hamieh M. Cunanan K. M. (2017). Targeting a CAR to the TRAC locus with CRISPR/Cas9 enhances tumour rejection. Nature 543, 113–117. 10.1038/nature21405 28225754 PMC5558614

[B34] De Felice D. Alaimo A. (2020). Mechanosensitive piezo channels in cancer: focus on altered calcium signaling in cancer cells and in tumor progression. Cancers (Basel) 12. 10.3390/cancers12071780 32635333 PMC7407875

[B35] Formenti S. C. Lee P. Adams S. Goldberg J.A.-O. Li X. Xie M. W. (2018). Focal irradiation and systemic TGFβ blockade in metastatic breast Cancer.Int J radiat *Oncol biol phys* . Clin. Cancer Res. 24 (11), 2493–2504. 10.1158/1078-0432.CCR-17-3322 29476019 PMC5999326

[B36] Fu W. Hou X. Ding L. Wei J. Hou W. (2025). Piezo1-related physiological and pathological processes in glioblastoma. Front. Cell Dev. Biol. 13, 1536320. 10.3389/fcell.2025.1536320 40061015 PMC11885286

[B37] Gensbittel V. Kräter M. Harlepp S. Busnelli I. Guck J. Goetz J. G. (2023). Mechanical adaptability of tumor cells in metastasis. Dev Cell. 56 (2), 164–179. 10.1016/j.devcel.2020.10.011 33238151

[B38] Ge J. Li W. Zhao Q. Li N. Chen M. Zhi P. (2015). Architecture of the mammalian mechanosensitive Piezo1 channel. Nature 527, 64–69. 10.1038/nature15247 26390154

[B39] Geng J. Zhao Q. Zhang T. Xiao B. (2017). In touch with the mechanosensitive Piezo channels: structure, ion permeation, and mechanotransduction. Curr. Top. Membr. 79, 159–195. 10.1016/bs.ctm.2016.11.006 28728816

[B40] Good C. R. Aznar M. A. Kuramitsu S. Samareh P. Agarwal S. Donahue G. (2021). An NK-like CAR-T cell transition in CAR-T cell dysfunction. Cell 184, 6081–6100.e6026. 10.1016/j.cell.2021.11.016 34861191 PMC8827167

[B41] Goswami R. Arya R. K. Sharma S. Dutta B. Stamov D. R. Zhu X. (2021). Mechanosensing by TRPV4 mediates stiffness-induced foreign body response and giant cell formation. Sci. Signal. 2 (14). 10.1126/scisignal.abd4077 34726952 PMC9976933

[B42] Greiner D. Xue Q. Waddell T. Q. Kurudza E. Chaudhary P. Belote R. L. (2023). Human CSPG4-targeting CAR-macrophages inhibit melanoma growth. Oncogene. 44 (22), 1665–1677. 10.1038/s41388-025-03332-0 40082557 PMC12122381

[B43] Guy C. Mitrea D. M. Chou P. C. Temirov J. Vignali K. M. Liu X. (2022). LAG3 associates with TCR-CD3 complexes and suppresses signaling by driving co-receptor-lck dissociation. Nat. Immunol. 23, 757–767. 10.1038/s41590-022-01176-4 35437325 PMC9106921

[B44] Hashimoto M. Araki K. Cardenas M. A. Li P. Jadhav R. R. Kissick H. T. (2022). PD-1 combination therapy with IL-2 modifies CD8(+) T cell exhaustion program. Nature 610, 173–181. 10.1038/s41586-022-05257-0 36171288 PMC9793890

[B45] Heldin C. H. Rubin K. Pietras K. Ostman A. (2004). High interstitial fluid pressure - an obstacle in cancer therapy. Nat. Rev. Cancer 4, 806–813. 10.1038/nrc1456 15510161

[B46] Hofmann M. Thimme R. Schamel W. W. (2024). PD-1 and LAG-3: synergistic fostering of T cell exhaustion. Signal Transduct. Target. Ther. 9, 291. 10.1038/s41392-024-02000-1 39424778 PMC11489778

[B47] Holt J.A.-O. Zeng W. Z. Evans E.A.-O. Woo S. H. Ma S. Abuwarda H. (2021). Spatiotemporal dynamics of Piezo1 localization controls keratinocyte migration during wound healing. eLife 10, e65415. 10.7554/eLife.65415 34569935 PMC8577841

[B48] Hong R. Yang D. Jing Y. Chen S. Tian H. Yang Y. (2023). Piezo1-Related physiological and pathological processes in CNS: focus on the gliomas. Cancers 15 (3), 883. 10.3390/cancers15030883 36765838 PMC9913778

[B49] Hope J. M. Dombroski J. A. Pereles R. S. Lopez-Cavestany M. Greenlee J. D. Schwager S. C. (2022). Fluid shear stress enhances T cell activation through Piezo1. BMC Biol. 20, 61. 10.1186/s12915-022-01266-7 35260156 PMC8904069

[B50] Hou B. Tang Y. Li W. Zeng Q. Chang D. (2019). Efficiency of CAR-T therapy for treatment of solid tumor in clinical trials: a meta-analysis. Dis. Markers 2019, 3425291. 10.1155/2019/3425291 30886654 PMC6388318

[B51] Jeffreys N. Brockman J. M. Zhai Y. Ingber D. E. Mooney D. J. (2024). Mechanical forces amplify TCR mechanotransduction in T cell activation and function. Appl. Phys. Rev. 11, 011304. 10.1063/5.0166848 38434676 PMC10848667

[B52] Jin Y. Zuo Y. Li G. Liu W. Pan Y. Fan T. (2024). Advances in spatial transcriptomics and its applications in cancer research. Mol. Cancer 23, 129. 10.1186/s12943-024-02040-9 38902727 PMC11188176

[B53] Jung P. Zhou X. Iden S. Bischoff M. Qu B. (2021). T cell stiffness is enhanced upon formation of immunological synapse. Elife 10. 10.7554/eLife.66643 34313220 PMC8360652

[B54] Jung I.-Y. Narayan V. McDonald S. Rech A. J. Bartoszek R. Hong G. (2023). BLIMP1 and NR4A3 transcription factors reciprocally regulate antitumor CAR T cell stemness and exhaustion. Sci Transl Med. 14 (670), eabn7336. 10.1126/scitranslmed.abn7336 36350986 PMC10257143

[B55] Jung Y. Han Y. Kang J. Yu S. L. Park S. R. (2025). Piezo1 selectively enhances TGF-β1-induced IgA class switching by B cells. Cell Mol. Life Sci. 82, 243. 10.1007/s00018-025-05789-4 40537609 PMC12179034

[B56] Kumari A. Veena S. M. Luha R. Tijore A.A.-O. (2023). Mechanobiological strategies to augment cancer treatment. ACS Omega 8 (45), 42072–42085. 10.1021/acsomega.3c06451 38024751 PMC10652740

[B57] Kalluri R. (2016). The biology and function of fibroblasts in cancer. Nat. Rev. Cancer 16, 582–598. 10.1038/nrc.2016.73 27550820

[B58] Kankeu Fonkoua L. A. Sirpilla O. Sakemura R. Siegler E. L. Kenderian S. S. (2022). CAR T cell therapy and the tumor microenvironment: current challenges and opportunities. Mol. Ther. Oncolytics 25, 69–77. 10.1016/j.omto.2022.03.009 35434273 PMC8980704

[B59] Khairullina Z. M. Vasileva V. Y. Chubinskiy-Nadezhdin V. I. (2025). Piezo1 ion channels regulate the Formation and spreading of human endometrial mesenchymal stem cell spheroids. Int. J. Mol. Sci. 26, 2474. 10.3390/ijms26062474 40141118 PMC11942067

[B60] Kim O.-H. Choi Y. W. Park J. H. Hong S. A. Hong M. Chang I. H. (2022). Fluid shear stress facilitates prostate cancer metastasis through Piezo1-Src-YAP axis. Life Sci. 308, 120936. 10.1016/j.lfs.2022.120936 36084759

[B61] Klabukov I. Kabakov A. E. Yakimova A. Baranovskii D. Sosin D. Atiakshin D. (2025). Tumor-Associated extracellular matrix obstacles for CAR-T cell therapy: approaches to overcoming. Curr. Oncol. 32 (2), 79. 10.3390/curroncol32020079 39996879 PMC11854105

[B62] Kuck L. McNamee A. P. Bordukova M. Sadafi A. Marr C. Peart J. N. (2024). Lysis of human erythrocytes due to Piezo1-dependent cytosolic calcium overload as a mechanism of circulatory removal. Proc. Natl. Acad. Sci. 121, e2407765121. 10.1073/pnas.2407765121 39207733 PMC11388408

[B63] Kuczek D. E. Larsen A. M. H. Thorseth M. L. Carretta M. Kalvisa A. Siersbæk M. S. (2019). Collagen density regulates the activity of tumor-infiltrating T cells. J. Immunother. Cancer 7, 68. 10.1186/s40425-019-0556-6 30867051 PMC6417085

[B64] Lai A. Cox C. D. Chandra Sekar N. Thurgood P. Jaworowski A. Peter K. (2022). Mechanosensing by Piezo1 and its implications for physiology and various pathologies. Biol. Rev. 97, 604–614. 10.1111/brv.12814 34781417

[B65] Laverty M. S. Benson B. L. Aguilar A. Kingsley D. Myers J. T. Choi S.-H. (2022). Mechanosensory channel Piezo1 contributes to T lymphocyte migration through direct interaction with integrins. J. Immunol. 208, 105.133. 10.4049/jimmunol.208.supp.105.33

[B66] Lebid A. Chung L. Pardoll D. M. Pan F. (2020). YAP Attenuates CD8 T cell-mediated anti-tumor response. Front. Immunol. 11, 580. 10.3389/fimmu.2020.00580 32322254 PMC7158852

[B67] Lebon D. Collet L. Djordjevic S. Gomila C. Ouled-Haddou H. Platon J. (2024). Piezo1 is essential for the survival and proliferation of acute myeloid leukemia cells. Cancer Med. 13, e6984. 10.1002/cam4.6984 38334477 PMC10854442

[B68] Leng S. Zhang X. Wang S. Qin J. Liu Q. Liu A. (2022). Ion channel Piezo1 activation promotes aerobic glycolysis in macrophages. Front. Immunol. 13, 976482. 10.3389/fimmu.2022.976482 36119083 PMC9479104

[B69] Leonardi M. V. Di Battista A. Donati L. Sforna L. Morena F. Di Cristina M. (2025). Piezo1 is an essential player in volume regulation of human glioblastoma cells. J. Physiol. 603, 4765–4784. 10.1113/JP289215 40838979 PMC12400783

[B70] Levental K. R. Yu H. Kass L. Lakins J. N. Egeblad M. Erler J. T. (2009). Matrix crosslinking forces tumor progression by enhancing integrin signaling. Cell 139, 891–906. 10.1016/j.cell.2009.10.027 19931152 PMC2788004

[B71] Li R. Ma C. Cai H. Chen W. (2020). The CAR T cell Mechanoimmunology at a glance. Adv. Sci. (Weinh) 7, 2002628. 10.1002/advs.202002628 33344135 PMC7740088

[B72] Liang T. Wang J. Yang Z. Zhang R. (2022). Comprehensive analysis of mRNA expression of Piezo1 and Piezo2 in tumor samples and their prognostic implications in gastric cancer. Discov. Oncol. 21 (1), 582. 10.1007/s12672-025-02309-5 40257604 PMC12011698

[B73] Liu C. S. C. Raychaudhuri D. Paul B. Chakrabarty Y. Ghosh A. R. Rahaman O. (2018). Cutting edge: Piezo1 mechanosensors optimize human T cell activation. J. Immunol. 200, 1255–1260. 10.4049/jimmunol.1701118 29330322

[B74] Liu G. Rui W. Zhao X. Lin X. (2021). Enhancing CAR-T cell efficacy in solid tumors by targeting the tumor microenvironment. Cell Mol. Immunol. 18, 1085–1095. 10.1038/s41423-021-00655-2 33785843 PMC8093220

[B75] Liu L. Qu Y. Cheng L. Yoon C. W. He P. Monther A. (2022). Engineering chimeric antigen receptor T cells for solid tumour therapy. Clin. Transl. Med. 12, e1141. 10.1002/ctm2.1141 36495108 PMC9736813

[B76] Liu L. Li Y. Li B. (2025). Interactions between cancer cells and tumor-associated macrophages in tumor microenvironment. Biochimica Biophysica Acta (BBA) - Rev. Cancer 1880, 189344. 10.1016/j.bbcan.2025.189344 40345263

[B77] Lüchtefeld I. Pivkin I. V. Gardini L. Zare-Eelanjegh E. Gäbelein C. Ihle S. J. (2024). Dissecting cell membrane tension dynamics and its effect on Piezo1-mediated cellular mechanosensitivity using force-controlled nanopipettes. Nat. Methods 21, 1063–1073. 10.1038/s41592-024-02277-8 38802520 PMC11166569

[B78] Luo Z. Yao X. Li M. Fang D. Fei Y. Cheng Z. (2022). Modulating tumor physical microenvironment for fueling CAR-T cell therapy. Adv. Drug Deliv. Rev. 185, 114301. 10.1016/j.addr.2022.114301 35439570

[B79] Luu N. Li R. Fang Y. Wang H. Song Y. Chen R. (2025). Piezo1-mediated mechano-energetics regulate CAR T cell function. Res. Sq [Preprint]. 10.21203/rs.3.rs-7776704/v1 41282104 PMC12633482

[B80] Lv J. Si T. Wang D. Zhang C. Zhou Y. Yu J. (2025). Mechanical signaling *via* β2 integrin decouples T cell proliferation and differentiation for generating stem cell-like CAR T cells. Immunity 58 (9), 2289–2304.e10. 10.1016/j.immuni.2025.07.018 40782798

[B81] Mai Z. Lin Y. Lin P. Zhao X. Cui L. (2024). Modulating extracellular matrix stiffness: a strategic approach to boost cancer immunotherapy. Cell Death Dis. 15, 307. 10.1038/s41419-024-06697-4 38693104 PMC11063215

[B82] Maneshi P. Mason J. Dongre M. Öhlund D. (2021). Targeting tumor-stromal interactions in pancreatic cancer: impact of collagens and mechanical traits. Front. Cell Dev. Biol. 9, 787485. 10.3389/fcell.2021.787485 34901028 PMC8656238

[B83] Mao J. Yang R. Yuan P. Wu F. Wei Y. Nie Y. (2023). Different stimuli induce endothelial dysfunction and promote atherosclerosis through the Piezo1/YAP signaling axis. Arch. Biochem. Biophys. 747, 109755. 10.1016/j.abb.2023.109755 37714252

[B84] Masuda H. (2025). Cancer-associated fibroblasts in cancer drug resistance and cancer progression: a review. Cell Death Discov. 11, 341. 10.1038/s41420-025-02566-x 40707476 PMC12289933

[B85] Mayorca-Guiliani A. E. Leeming D. J. Henriksen K. Mortensen J. H. Nielsen S. H. Anstee Q. M. (2025). ECM formation and degradation during fibrosis, repair, and regeneration. Npj Metabolic Health Dis. 3, 25. 10.1038/s44324-025-00063-4 40604328 PMC12441159

[B86] Mazloomi M. Doustmihan A. Alimohammadvand S. Hamishehkar H. Hamblin M. R. Jahanban Esfahlan R. (2025). Advanced drug delivery platforms target cancer stem cells. Asian J. Pharm. Sci. 20, 101036. 10.1016/j.ajps.2025.101036 40503056 PMC12152564

[B87] Mei J. Chu J. Yang K. Luo Z. Yang J. Xu J. (2024). Angiotensin receptor blocker attacks armored and cold tumors and boosts immune checkpoint blockade. J. Immunother. Cancer 12, e009327. 10.1136/jitc-2024-009327 39244215 PMC11418576

[B88] Meng K. P. Majedi F. S. Thauland T. J. Butte M. J. (2020). Mechanosensing through YAP controls T cell activation and metabolism. J. Exp. Med. 217. 10.1084/jem.20200053 32484502 PMC7398163

[B89] Nagase T. Nagase M. (2024). Piezo ion channels: long-sought-after mechanosensors mediating hypertension and hypertensive nephropathy. Hypertens. Res. 47, 2786–2799. 10.1038/s41440-024-01820-6 39103520

[B90] Neophytou C. Stylianopoulos T. Mpekris F. (2025). The synergistic potential of mechanotherapy and sonopermeation to enhance cancer treatment effectiveness. Npj Biol. Phys. Mech. 2, 13. 10.1038/s44341-025-00017-3 40337117 PMC12052595

[B91] Nia H. T. Liu H. Seano G. Datta M. Jones D. Rahbari N. (2016). Solid stress and elastic energy as measures of tumour mechanopathology. Nat. Biomed. Eng. 1, 0004. 10.1038/s41551-016-0004 28966873 PMC5621647

[B92] Nia H. T. Munn L. L. Jain R. K. (2020). Physical traits of cancer. Science 370. 10.1126/science.aaz0868 33122355 PMC8274378

[B93] Nikoo M. Rudiansyah M. Bokov D. O. Jainakbaev N. T. Suksatan W.A.-O. Ansari M. J. (2024). Potential of chimeric antigen receptor (CAR)-redirected immune cells in breast cancer therapies: recent advances. J Cell Mol Med. 26 (15), 4137–4156. 10.1111/jcmm.17465 35762299 PMC9344815

[B94] Nilius B. Honoré E. (2012). Sensing pressure with ion channels. Trends Neurosci. 35, 477–486. 10.1016/j.tins.2012.04.002 22622029

[B95] Niu B. Zhou F. Su Y. Wang L. Xu Y. Yi Z. (2019). Different expression characteristics of LAG3 and PD-1 in sepsis and their synergistic effect on T cell exhaustion: a new strategy for immune checkpoint blockade. Front. Immunol. 10, 1888. 10.3389/fimmu.2019.01888 31440257 PMC6693426

[B96] Niu W. Liu X. Deng B. Hong T. Wang C. Yan Y. (2025). Piezo1 deletion mitigates diabetic cardiomyopathy by maintaining mitochondrial dynamics *via* ERK/Drp1 pathway. Cardiovasc Diabetol. 24, 127. 10.1186/s12933-025-02625-8 40114130 PMC11927149

[B97] Opazo P. Viana da Silva S. Carta M. Breillat C. Coultrap S. J. Grillo-Bosch D. (2018). CaMKII metaplasticity drives Aβ oligomer-mediated synaptotoxicity. Cell Rep. 23 (11), 3137–3145. 10.1016/j.celrep.2018.05.036 29898386 PMC6089247

[B98] Pang R. Sun W. Yang Y. Wen D. Lin F. Wang D. (2024). Piezo1 mechanically regulates the antitumour cytotoxicity of T lymphocytes. Nat. Biomed. Eng. 8, 1162–1176. 10.1038/s41551-024-01188-5 38514773

[B99] Papavassiliou K. A. Basdra E. K. Papavassiliou A. G. (2023). The emerging promise of tumour mechanobiology in cancer treatment. Eur. J. Cancer, 190, 112938. 10.1016/j.ejca.2023.112938 37390803

[B100] Park H. E. Jo S. H. Lee R. H. Macks C. P. Ku T. Park J. (2023). Spatial transcriptomics: technical aspects of recent developments and their applications in neuroscience and cancer research. Adv. Sci. (Weinh) 10, e2206939. 10.1002/advs.202206939 37026425 PMC10238226

[B101] Pavel M. Renna M. Park S. J. Menzies F. M. Ricketts T. Füllgrabe J. (2018). Contact inhibition controls cell survival and proliferation *via* YAP/TAZ-autophagy axis. Nat. Commun. 9, 2961. 10.1038/s41467-018-05388-x 30054475 PMC6063886

[B102] Plodinec M. Loparic M. Monnier C. A. Obermann E. C. Zanetti-Dallenbach R. Oertle P. (2012). The nanomechanical signature of breast cancer. Nat. Nanotechnol. 7, 757–765. 10.1038/nnano.2012.167 23085644

[B103] Qi M. Cattaneo G. Camillo C. Quattrocchi E. Zhang L. Tejeda-Polanco E. (2025). Pitfalls and strategies of CAR-T therapy in solid tumors and implications for chordoma treatment. Immunotherapy 17, 735–747. 10.1080/1750743X.2025.2536458 40782028 PMC12355681

[B104] Qiu Z. Guo J. Kala S. Zhu J. Xian Q. Qiu W. (2019). The mechanosensitive ion channel Piezo1 significantly mediates *in vitro* ultrasonic stimulation of neurons. iScience 21, 448–457. 10.1016/j.isci.2019.10.037 31707258 PMC6849147

[B105] Qu P. Zhang H. (2025). The dual role of Piezo1 in tumor cells and immune cells: a new target for cancer therapy. Front. Immunol. 16, 1635388. 10.3389/fimmu.2025.1635388 40821847 PMC12350401

[B106] Raju G. S. R. Pavitra E. Varaprasad G. L. Bandaru S. S. Nagaraju G. P. Farran B. (2022). Nanoparticles mediated tumor microenvironment modulation: current advances and applications. J. Nanobiotechnology 20, 274. 10.1186/s12951-022-01476-9 35701781 PMC9195263

[B107] Ren X. Zhuang H. Li B. Jiang F. Zhang Y. Zhou P. (2023). Gsmtx4 alleviated osteoarthritis through Piezo1/Calcineurin/NFAT1 signaling axis under excessive mechanical strain. Int. J. Mol. Sci. 24, 4022. 10.3390/ijms24044022 36835440 PMC9961447

[B108] Revilla S. A. Cutilli A. Cambiaso E. Rockx-Brouwer D. Frederiks C. L. Falandt M. (2025). Impact of 3D cell culture hydrogels derived from basement membrane extracts or nanofibrillar cellulose on CAR-T cell activation. iScience 28, 113234. 10.1016/j.isci.2025.113234 40837221 PMC12362413

[B109] Roy N. H. MacKay J. L. Robertson T. F. Hammer D. A. Burkhardt J. K. (2018). Crk adaptor proteins mediate actin-dependent T cell migration and mechanosensing induced by the integrin LFA-1. Sci. Signal. 11, eaat3178. 10.1126/scisignal.aat3178 30538176 PMC6333317

[B110] Salavati H. Debbaut C. Pullens P. Ceelen W. (2022). Interstitial fluid pressure as an emerging biomarker in solid tumors. Biochim. Biophys. Acta Rev. Cancer 1877, 188792. 10.1016/j.bbcan.2022.188792 36084861

[B111] Saotome K. Murthy S. E. Kefauver J. M. Whitwam T. Patapoutian A. Ward A. B. (2018). Structure of the mechanically activated ion channel Piezo1. Nature 554, 481–486. 10.1038/nature25453 29261642 PMC6010196

[B112] Scheraga R. G. Southern B. D. Grove L. M. Olman M. A. (2020). The role of TRPV4 in regulating innate immune cell function in lung inflammation. Front. Immunol. 11, 11–2020. 10.3389/fimmu.2020.01211 32676078 PMC7333351

[B113] Schurich A. Magalhaes I. Mattsson J. (2019). Metabolic regulation of CAR T cell function by the hypoxic microenvironment in solid tumors. Immunotherapy, 11 (4), 335–345. 10.2217/imt-2018-0141 30678555

[B114] Schuster S. J. Svoboda J. Chong E. A. Nasta S. D. Mato A. R. Anak Ö. (2017). Chimeric antigen receptor T cells in refractory B-Cell lymphomas. N. Engl. J. Med. 377, 2545–2554. 10.1056/NEJMoa1708566 29226764 PMC5788566

[B115] Shen H. Ojo O. A. Ding H. Mullen L. J. Xing C. Hossain M. I. (2024). HIF1α-regulated glycolysis promotes activation-induced cell death and IFN-γ induction in hypoxic T cells. Nat. Commun. 15, 9394. 10.1038/s41467-024-53593-8 39477954 PMC11526104

[B116] Shi X. Yang J. Deng S. Xu H. Wu D. Zeng Q. (2022). TGF-β signaling in the tumor metabolic microenvironment and targeted therapies. J. Hematol. and Oncol. 15, 135. 10.1186/s13045-022-01349-6 36115986 PMC9482317

[B117] Siegel R. L. Miller K. D. Wagle N. S. Jemal A. (2023). Cancer statistics. CA A Cancer J. Clin. 73, 17–48. 10.3322/caac.21332 36633525

[B118] Smith H. A. Kang Y. (2013). The metastasis-promoting roles of tumor-associated immune cells. J. Mol. Med. Berl. 91, 411–429. 10.1007/s00109-013-1021-5 23515621 PMC3697909

[B119] Solis A. G. Bielecki P. Steach H. R. Sharma L. Harman C. C. D. Yun S. (2019). Mechanosensation of cyclical force by Piezo1 is essential for innate immunity. Nature 573, 69–74. 10.1038/s41586-019-1485-8 31435009 PMC6939392

[B120] Song Y. Li D. Farrelly O. Miles L. Li F. Kim S. E. (2019). The mechanosensitive ion channel piezo inhibits axon regeneration. Neuron 102, 373–389.e376. 10.1016/j.neuron.2019.01.050 30819546 PMC6487666

[B121] Starostina I. Jang Y.-K. Kim H.-S. Suh J.-S. Ahn S.-H. Choi G.-H. (2021). Distinct calcium regulation of TRPM7 mechanosensitive channels at plasma membrane microdomains visualized by FRET-based single cell imaging. Sci. Rep. 11, 17893. 10.1038/s41598-021-97326-z 34504177 PMC8429465

[B122] Sun L. Wang Y. Kan T. Wang H. Cui J. Wang L. (2025). Elevated expression of Piezo1 activates the cGAS-STING pathway in chondrocytes by releasing mitochondrial DNA. Osteoarthr. Cartil. 33, 601–615. 10.1016/j.joca.2025.02.778 39978573

[B123] Swain S. M. Romac J. M. Vigna S. R. Liddle R. A. (2022). Piezo1-mediated stellate cell activation causes pressure-induced pancreatic fibrosis in mice. JCI Insight 7 (8), e158288. 10.1172/jci.insight.158288 35451372 PMC9089793

[B124] Tabdanov E. D. Rodríguez-Merced N. J. Cartagena-Rivera A. X. Puram V. V. Callaway M. K. Ensminger E. A. (2021). Engineering T cells to enhance 3D migration through structurally and mechanically complex tumor microenvironments. Nat. Commun. 12, 2815. 10.1038/s41467-021-22985-5 33990566 PMC8121808

[B125] Tang L. Dai F. Liu Y. Yu X. Huang C. Wang Y. (2018). RhoA/ROCK signaling regulates smooth muscle phenotypic modulation and vascular remodeling *via* the JNK pathway and vimentin cytoskeleton. Pharmacol. Res. 133, 201–212. 10.1016/j.phrs.2018.05.011 29791873

[B126] Tang H. Zeng R. He E. Zhang I. Ding C. Zhang A. (2022). Piezo-Type mechanosensitive ion channel component 1 (Piezo1): a promising therapeutic target and its modulators. J. Med. Chem. 65, 6441–6453. 10.1021/acs.jmedchem.2c00085 35466678

[B128] Tomida T. Hirose K. Takizawa A. Shibasaki F. Iino M. (2003). NFAT functions as a working memory of Ca2+ signals in decoding Ca2+ oscillation. EMBO J. 22 (15), 3825–3832. 10.1093/emboj/cdg381 12881417 PMC169054

[B129] Vaeth M. Eckstein M. Shaw P. J. Kozhaya L. Yang J. Berberich-Siebelt F. (2016). Store-Operated Ca(^2+^) entry in follicular T cells controls humoral immune responses and autoimmunity. Immunity 44, 1350–1364. 10.1016/j.immuni.2016.04.013 27261277 PMC4917422

[B130] Vasileva V. Morachevskaya E. Sudarikova A. Negulyaev Y. Chubinskiy-Nadezhdin V. (2021). Selective chemical activation of Piezo1 in leukemia cell membrane: single channel analysis. Int. J. Mol. Sci. 22, 7839. 10.3390/ijms22157839 34360605 PMC8346046

[B131] De Vecchis D. Beech D. J. Kalli A. C. (2021). Molecular dynamics simulations of Piezo1 channel opening by increases in membrane tension. Biophys. J. 120, 1510–1521. 10.1016/j.bpj.2021.02.006 33582135 PMC8105709

[B132] de Visser K. E. Joyce J. A. (2023). The evolving tumor microenvironment: from cancer initiation to metastatic outgrowth. Cancer Cell 41, 374–403. 10.1016/j.ccell.2023.02.016 36917948

[B133] Wang X. Freire Valls A. Schermann G. Shen Y. Moya I. M. Castro L. (2017). YAP/TAZ orchestrate VEGF signaling during developmental angiogenesis. Dev. Cell 42, 462–478.e467. 10.1016/j.devcel.2017.08.002 28867486

[B134] Wang L. Zhou H. Zhang M. Liu W. Deng T. Zhao Q. (2019). Structure and mechanogating of the mammalian tactile channel Piezo2. Nature 573, 225–229. 10.1038/s41586-019-1505-8 31435011

[B135] Wang Y. Drum D. L. Sun R. Zhang Y. Yu L. Jia L. (2023). Stressed target cancer cells drive nongenetic reprogramming of CAR T cells and solid tumor microenvironment. Nat Commun. 14 (1), 5727. 10.1038/s41467-023-41282-x 37714830 PMC10504259

[B136] Wang J. Jing F. Zhao Y. You Z. Zhang A. Qin S. (2025). Piezo1: structural pharmacology and mechanotransduction mechanisms. Trends Pharmacol. Sci. 46, 752–770. 10.1016/j.tips.2025.06.009 40750459

[B137] Watanabe K. Kuramitsu S. Posey A. D. Jr. June C. H. (2018). Expanding the therapeutic window for CAR T cell therapy in solid tumors: the knowns and unknowns of CAR T cell biology. Front. Immunol. 9, 2486. 10.3389/fimmu.2018.02486 30416506 PMC6212550

[B138] Wei J. Han X. Bo J. Han W. (2019). Target selection for CAR-T therapy. J. Hematol. and Oncol. 12, 62. 10.1186/s13045-019-0758-x 31221182 PMC6587237

[B139] Wherry E. J. Kurachi M. (2015). Molecular and cellular insights into T cell exhaustion. Nat. Rev. Immunol. 15, 486–499. 10.1038/nri3862 26205583 PMC4889009

[B140] Wolf K. Te Lindert M. Krause M. Alexander S. Te Riet J. Willis A. L. (2013). Physical limits of cell migration: control by ECM space and nuclear deformation and tuning by proteolysis and traction force. J. Cell Biol. 201, 1069–1084. 10.1083/jcb.201210152 23798731 PMC3691458

[B141] Woo S. H. Lukacs V. de Nooij J. C. Zaytseva D. Criddle C. R. Francisco A. (2015). Piezo2 is the principal mechanotransduction channel for proprioception. Nat. Neurosci. 18, 1756–1762. 10.1038/nn.4162 26551544 PMC4661126

[B142] Wu X. Ye Z. (2025). Mechanoimmunology of T cell Activation. Scand. J. Immunol. 101, e70009. 10.1111/sji.70009 39973081

[B143] Wu J. Lewis A. H. Grandl J. T. (2017). Tension, and transduction - the function and regulation of piezo ion channels. Trends Biochem. Sci. 42, 57–71. 10.1016/j.tibs.2016.09.004 27743844 PMC5407468

[B144] Wu B. Liu D.-A. Guan L. Myint P. K. Chin L. Dang H. (2023a). Stiff matrix induces exosome secretion to promote tumour growth. Nat. Cell Biol. 25, 415–424. 10.1038/s41556-023-01092-1 36797475 PMC10351222

[B145] Wu W. Wang X. Liao L. Chen J. Wang Y. Yao M. (2023b). The TRPM7 channel reprograms cellular glycolysis to drive tumorigenesis and angiogenesis. Cell Death and Dis. 14, 183. 10.1038/s41419-023-05701-7 36878949 PMC9988972

[B146] Xu H. He Y. Hong T. Bi C. Li J. Xia M. (2022). Piezo1 in vascular remodeling of atherosclerosis and pulmonary arterial hypertension: a potential therapeutic target. Front. Cardiovasc. Med. 9, 1021540. 10.3389/fcvm.2022.1021540 36247424 PMC9557227

[B147] Xu L. Li T. Cao Y. He Y. Shao Z. Liu S. (2025). Piezo1 mediates periostin+ myofibroblast activation and pulmonary fibrosis in mice. J. Clin. Invest 135, e184158. 10.1172/JCI184158 40454481 PMC12126248

[B148] Yamazaki T. Gunderson A. J. Gilchrist M. Whiteford M. Kiely M. X. Hayman A. (2022). Galunisertib plus neoadjuvant chemoradiotherapy in patients with locally advanced rectal cancer: a single-arm, phase 2 trial. Lancet Oncol. 23, 1189–1200. 10.1016/S1470-2045(22)00446-6 35952709

[B149] Yang Y. Wang D. Zhang C. Yang W. Li C. Gao Z. (2022). Piezo1 mediates endothelial atherogenic inflammatory responses *via* regulation of YAP/TAZ activation. Hum Cell. 35 (1), 51–62. 10.1007/s13577-021-00600-5 34606042

[B150] Yang C. Dong X. Sun B. Cao T. Xie R. Zhang Y. (2024). Physical immune escape: weakened mechanical communication leads to escape of metastatic colorectal carcinoma cells from macrophages. Proc. Natl. Acad. Sci. U. S. A. 121, e2322479121. 10.1073/pnas.2322479121 38771871 PMC11145255

[B151] Yang Z. Liu X. Zhu J. Chai Y. Cong B. Li B. (2025). Inhibiting intracellular CD28 in cancer cells enhances antitumor immunity and overcomes anti-PD-1 resistance *via* targeting PD-L1. Cancer Cell 43, 86–102.e110. 10.1016/j.ccell.2024.11.008 39672166

[B152] Yin X. Ding Z. Yu L. Zhang X. Gao Y. Li Y. (2025). Orchestrating intratumoral DC-T cell immunity for enhanced tumor control *via* radiotherapy-activated TLR7/8 prodrugs in mice. Nat. Commun. 16, 6020. 10.1038/s41467-025-60769-3 40592817 PMC12216172

[B153] Yu J.-L. Liao H.-Y. (2021). Piezo-type mechanosensitive ion channel component 1 (Piezo1) in human cancer. Biomed. and Pharmacother. 140, 111692. 10.1016/j.biopha.2021.111692 34004511

[B154] Yu Y. Wu X. Wang M. Liu W. Zhang L. Zhang Y. (2022). Optogenetic-controlled immunotherapeutic designer cells for post-surgical cancer immunotherapy. Nat. Commun. 13, 6357. 10.1038/s41467-022-33891-9 36289204 PMC9605972

[B155] Zanconato F. Cordenonsi M. Piccolo S. (2016). YAP/TAZ at the roots of cancer. Cancer Cell 29, 783–803. 10.1016/j.ccell.2016.05.005 27300434 PMC6186419

[B156] Zebley C. C. Zehn D. Gottschalk S. Chi H. (2024). T cell dysfunction and therapeutic intervention in cancer. Nat. Immunol. 25, 1344–1354. 10.1038/s41590-024-01896-9 39025962 PMC11616736

[B157] Zhang Y. Su S.-a. Li W. Ma Y. Shen J. Wang Y. (2021). Piezo1-Mediated mechanotransduction promotes cardiac hypertrophy by impairing calcium homeostasis to activate Calpain/Calcineurin signaling. Hypertension 78, 647–660. 10.1161/HYPERTENSIONAHA.121.17177 34333987

[B158] Zhang X. Al-Danakh A. Zhu X. Feng D. Yang L. Wu H. (2025). Insights into the mechanisms, regulation, and therapeutic implications of extracellular matrix stiffness in cancer. Bioeng. Transl. Med. 10, e10698. 10.1002/btm2.10698 39801760 PMC11711218

[B159] Zhao Q. Zhou H. Chi S. Wang Y. Wang J. Geng J. (2018). Structure and mechanogating mechanism of the Piezo1 channel. Nature 554, 487–492. 10.1038/nature25743 29469092

[B160] Zhao R. Zhou X. Khan E. S. Alansary D. Friedmann K. S. Yang W. (2021). Targeting the microtubule-network rescues CTL killing efficiency in dense 3D matrices. Front. Immunol. 12, 729820. 10.3389/fimmu.2021.729820 34484240 PMC8416057

[B161] Zhao Z. Li Q. Qu C. Jiang Z. Jia G. Lan G. (2025). A collagenase nanogel backpack improves CAR-T cell therapy outcomes in pancreatic cancer. Nat. Nanotechnol. 20, 1131–1141. 10.1038/s41565-025-01924-1 40389641

[B162] Zheng R. Shen K. Liang S. Lyu Y. Zhang S. Dong H. (2024). Specific ECM degradation potentiates the antitumor activity of CAR-T cells in solid tumors. Cell. and Mol. Immunol. 21, 1491–1504. 10.1038/s41423-024-01228-9 39472748 PMC11606952

[B163] Zhu X. Li Q. Zhu X. (2022). Mechanisms of CAR T cell exhaustion and current counteraction strategies. Front. Cell Dev. Biol. 10, 1034257. 10.3389/fcell.2022.1034257 36568989 PMC9773844

[B164] Zhu Y. Feng J. Wan R. Huang W. (2023). CAR T cell therapy: remedies of Current challenges in design, injection, infiltration and working. Drug Des. Devel Ther. 17, 1783–1792. 10.2147/DDDT.S413348 37337518 PMC10277020

[B165] Zhu Y. Chen J. Chen C. Tang R. Xu J. Shi S. (2025). Deciphering mechanical cues in the microenvironment: from non-malignant settings to tumor progression. Biomark. Res. 13, 11. 10.1186/s40364-025-00727-9 39849659 PMC11755887

[B166] Zhuang C. Gould J. E. Enninful A. Shao S. Mak M. (2023). Biophysical and mechanobiological considerations for T cell-based immunotherapy. Trends Pharmacol. Sci. 44, 366–378. 10.1016/j.tips.2023.03.007 37172572 PMC10188210

